# Advancing Genetic Selection and Behavioral Genomics of Working Dogs Through Collaborative Science

**DOI:** 10.3389/fvets.2021.662429

**Published:** 2021-09-06

**Authors:** Frances L. Chen, Madeline Zimmermann, Jessica P. Hekman, Kathryn A. Lord, Brittney Logan, Jane Russenberger, Eldin A. Leighton, Elinor K. Karlsson

**Affiliations:** ^1^Vertebrate Genomics, Broad Institute of MIT and Harvard, Cambridge, MA, United States; ^2^Cellular Longevity, Inc., San Francisco, CA, United States; ^3^Guiding Eyes for the Blind, Yorktown Heights, NY, United States; ^4^Bioinformatics and Integrative Biology, University of Massachusetts Medical School, Worcester, MA, United States; ^5^International Working Dog Breeding Association, San Antonio, TX, United States; ^6^Canine Genetic Services, LLC, Watertown, CT, United States; ^7^Darwin's Ark Foundation, Seattle, WA, United States

**Keywords:** dog breeding, genetic selection, behavior, genomics, heritability, EBV, working dog, guide dog

## Abstract

The ancient partnership between people and dogs is struggling to meet modern day needs, with demand exceeding our capacity to safely breed high-performing and healthy dogs. New statistical genetic approaches and genomic technology have the potential to revolutionize dog breeding, by transitioning from problematic phenotypic selection to methods that can preserve genetic diversity while increasing the proportion of successful dogs. To fully utilize this technology will require ultra large datasets, with hundreds of thousands of dogs. Today, dog breeders struggle to apply even the tools available now, stymied by the need for sophisticated data storage infrastructure and expertise in statistical genetics. Here, we review recent advances in animal breeding, and how a new approach to dog breeding would address the needs of working dog breeders today while also providing them with a path to realizing the next generation of technology. We provide a step-by-step guide for dog breeders to start implementing estimated breeding value selection in their programs now, and we describe how genotyping and DNA sequencing data, as it becomes more widely available, can be integrated into this approach. Finally, we call for data sharing among dog breeding programs as a path to achieving a future that can benefit all dogs, and their human partners too.

## Introduction

**Figure 1:**
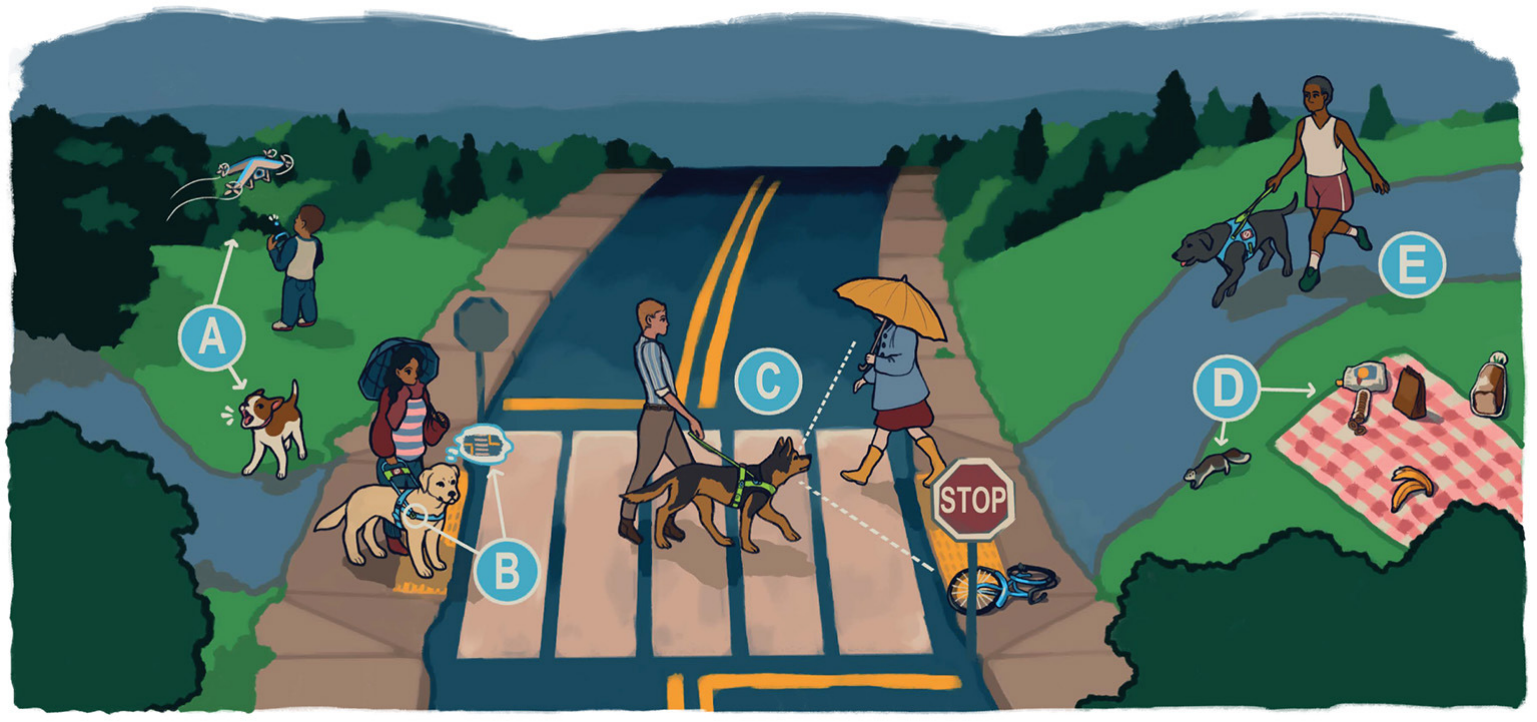
Skills required of a high-performing guide dog. A high performing working dog is required to fulfill a demanding set of criteria that vary by working dog type. While some, such as resilience, are required for nearly all working dogs, other skills are job-specific. To illustrate this, we describe some of the major requirements for a guide dog, the working dog type bred by Guiding Eyes for the Blind. **(A)** A guide dog must not be frightened of or bark at things that typically alarm other dogs. When working, they must ignore distractions such as other dogs or other animals around them. **(B)** They must be comfortable leading out with a steady pace and pull when working in harness, while also remaining calm and focused in all situations. They must learn a wide range of commands, but also be able to ignore commands when they are not safe, and problem-solve when a command is not possible. **(C)** When there are obstacles or dangers in the handler's path, a guide dog must alert the handler by stopping, or navigating their handler around the obstacles, and then resume walking in the target direction. When working, they should not be distracted by other people. **(D)** A guide dog must resist chasing things while working, and ignore enticing scents, including food. **(E)** A guide dog needs to be physically healthy, and matched to the stride and personality of their handler. Image credit: Kathleen Morrill.

A successful working dog is healthy, physically fit, and able to perform at an exceptionally high-level, with the behavioral, physiological, and structural characteristics required varying by job (1) (Figure 1). Over the past 20 years, especially since the attacks of September 11, 2001, the demand for high-quality working dogs around the world has soared, while the supply of these dogs has either remained unchanged or declined, resulting in increasing costs even as the quality of the dogs has suffered (2–4). With rates of visual impairment and blindness in the United States anticipated to double by 2050 as populations age, requests for guide dogs, already often difficult to access (5), will almost certainly increase further (6). To meet this increasing demand, organizations that breed working dogs need to use scientifically proven, modern breeding best practices that can increase the production of high-performing, healthy dogs (7).

Working dogs, in various forms and with various functions, have been part of human societies for thousands of years. Sled dogs were used in Eastern Siberia over 9,000 years ago (8, 9), and ancient Romans had both livestock guarding dogs and hunting dogs (10, 11). During this time, humans likely exerted ***postzygotic selection*** by favoring the highest performing dogs, increasing the prevalence of desirable traits among their offspring. Compared to modern dog breeds, ancient working dog populations were outbred and genetically diverse. Modern dog breeding started in the mid 1800s, and historical records and genomic studies suggest modern dog breeders predominantly favored form and pedigree over function (12, 13). The genomic loci most differentiated between breeds have been implicated in physical traits like body size, coat characteristics, and ear shape. While all dogs in a modern dog breed may look similar to one another, behavior and personality is highly variable.

Because the genetic variants that confer working dog traits predate modern breeds, any dog, purebred or not, may, by chance, inherit the genetic profile of a high-performing working dog, although this probability may vary depending on the dog's breed ancestry. The goal of selective breeding is to increase the average ***genetic merit*** of a population, thereby increasing the likelihood that, in the next generation, more dogs will be higher-performing than dogs in the current generation. To reach this goal, successful breeding programs will also attempt to optimize the environment in early puppyhood for long-term success (14–17).

Implementing a successful working dog breeding program is enormously challenging because of the complexity of the traits themselves, and the risk of inbreeding and other inadvertent consequences. Behavioral traits, difficult to breed for because of their genetic complexity and environmental malleability, may also be the most critical (18, 19). If not done with care, selective breeding can significantly reduce genetic diversity of the population, leading to increased rates of disease and shortening lifespans (20). While using dogs from outside populations as breeders can restore diversity, it risks lowering the success rate of the importing breeding program for generations, if the imported breeder is of lower genetic merit. Even successful selection programs can have unexpected consequences. Selection for dogs that are easy to control, for example, may increase the rate of excessive body sensitivity.

To address the increasing demand for working dogs, canine breeding programs need to utilize modern animal breeding practices, including cutting-edge, and rapidly advancing, genomic technologies. Here, we provide a roadmap for implementing a modern canine breeding program, and describe the synergistic collaboration between two non-profit projects to support dog breeders transitioning from time-worn techniques to modern, scientifically proven methods. The ***International Working Dog Registry (IWDR)*** is a centralized database that already contains uniformly coded records on over 64,000 dogs. Uniformity in coding is accomplished using drop-down lists of coding choices, from which one must be chosen, with very little free-text permitted anywhere in a dog's record. IWDR implements modern animal breeding tools within the registry, and it supports training for breeders seeking to employ genetic selection. The ***Working Dog Project*** is an open-science initiative for working dog genomics, designed to engage tens of thousands of dogs in research studies to develop the next generation of genomic and medical technology for dogs. Working together, the two aim to increase the supply of high-quality working dogs, while supporting research to improve the health and welfare of all dogs.

## Breeding Working Dogs

Managers of working dog breeding programs face the daunting challenge of producing large numbers of puppies, often over 100 per year, while maintaining, or even increasing, the percentage of successful dogs. There is almost always at least one plausible reason not to breed a young female, or to avoid a particular mate, especially when the goal is to avoid producing any disease-affected puppies, but this has to be balanced against the need to produce puppies that easily develop into behaviorally appropriate adult dogs. To balance these competing forces, a production plan needs to be followed that includes an objective method for choosing young dogs to become parents of the next generation. By following this plan, selection will change allele frequencies in the population, and the puppies produced in each successive generation will be healthier and endowed with more desirable behaviors than those of their parents. This objective, science-driven approach, proven to work by large guide dog breeding programs (7), has clear advantages over today's most common approaches to dog breeding.

At conception, each future puppy inherits its genetic foundation (***genotype***) from its parents. The local environment in which each genotype develops into a working adult has the potential to mold and shape that genotype in a myriad of ways. The ultimate challenge of dog breeding is to wisely use observed ***phenotypes*** to accurately predict the non-observable underlying genotypes.

For centuries, dog breeders have used ***phenotypic selection*** to influence observable traits or behaviors seen in a population. This traditional process, where dogs are chosen to become parents based on their individual phenotypes, has slowly molded and shaped ancestral dogs into today's modern breeds (21, 22). Using this process to produce genetic change, however, is very challenging because a dog's own phenotype is often a rather poor predictor of the dog's true genetic merit or genotype. Scientifically advanced working dog breeding programs of today utilize a data-driven method called ***estimated breeding values*** ***(EBVs)*** for meeting this challenge. It incorporates statistics and phenotypes to more accurately identify young dogs to be kept for breeding, even before producing their first litter.

Just as in phenotypic selection, EBVs rely on trait measurements made on individual dogs, but the calculation process is objective, deterministic, and grounded in modern statistical prediction theory. Furthermore, many people can learn how to use EBVs, even if they do not fully understand the process by which EBVs are calculated. For molecular geneticists, it may be helpful to know that EBVs in the context of this paper are very similar in concept to ***polygenic risk scores*** in human genetics (23), but with one fundamental difference. In the animal breeding world, the family structure of most populations includes rather large half-sib and full-sib families. The process for producing EBVs takes this family pedigree structure into account.

Since the 1940s, livestock breeders have used some form of EBVs and genetic selection to obtain genetic change in economically important production traits (24, 25). For example, breeders of American Angus cattle increased average weaning weight of bull calves by about 4 pounds per year between 1972 and 2021 (26). Similarly, between 2000 and 2016, US dairy cattle breeders, by applying selection pressure to increase the productive life, achieved an increase of about 10 months (27). Using exactly the same techniques as the livestock breeders, the dog breeding program at The Seeing Eye improved trainability for working as a guide while reducing the frequency of phenotypes that impact working longevity, including poor hip quality (7). After eight generations of selection, the percentage of dogs with an excellent hip quality score (as assessed by an extended view hip score) increased from 34 to 93% in German Shepherd Dogs and from 43 to 94% in Labrador retrievers.

Phenotype selection, when carefully implemented, can be effective for altering the prevalence of single traits. In Sweden, phenotype selection alone reduced rates of moderate to severe hip dysplasia in at-risk breeds by one third (28). However, genetic selection results in more improvement than phenotype selection (29), allows for continuing improvement even after phenotype selection has reduced the frequency of undesirable characteristics, and makes it possible to select on multiple traits in parallel (28).

The advent of inexpensive whole-genome genotyping and sequencing technology could allow relative genetic merit to be predicted more accurately from genotype in the future (23). These powerful approaches are not yet possible, but offer the potential to further improve on EBV selection.

## Best Practices for Genetic Improvement in a Breeding Population

Working dog programs and breeders using only phenotype selection will find it difficult, if not impossible, to maintain and improve the health and performance of their dogs over many generations. The vast majority of traits are complex, with tens or hundreds of different genes shaping a dog's inherited genetic potential, which is further influenced by their environment. To increase the frequency of a phenotype in a population, dogs should be selected for breeding based on the likelihood that their progeny will exhibit that phenotype. While adult phenotype is a reasonable proxy for this likelihood for a simple genetic trait, like coat color or hair type, for ***complex traits*** (e.g., behavior, or susceptibility to diseases like cancer) the correlation is much less clear.

Using phenotype selection, genetic improvement will be, at best, slow. Selecting dogs for breeding based solely on their adult phenotype is an inefficient way to increase the frequency of polygenically inherited desired traits in the next generation and is likely to lead to reduced genetic diversity and increased rates of disease (20, 30, 31). Breeders will often invest significant time and resources in attempting to identify genetically superior dogs by studying pedigree databases for evidence of the desired trait in related dogs, but this approach lacks a systematic means of ranking an individual's genetic merit based on its family relationships.

EBV selection is far more powerful than phenotype selection. The EBV for a given trait on a specific dog is calculated using phenotype data, both from that dog and from all related dogs in the population, when those data are available. Because most breeding plans are focused on improving more than one trait, each dog will have a set of EBVs, one for each trait. This set of EBVs for multiple traits can then be combined into one overall ***selection index*** that weights each trait based on its importance to the breeding program (32–34). This overall selection index value then reflects each young dog's overall relative genetic merit, which is also an indication of the dog's ability to produce offspring with all the phenotypes included in the breeding goal. By using an overall selection index, breeding program managers can identify a genetically diverse cohort of young dogs most likely to confer desired traits in their offspring.

Despite its utility, EBV selection has not been widely used in dog breeding because it requires large, accurate pedigrees and phenotypes assembled into one uniformly coded database, as well as expertise in statistical genetics and data processing. These requirements have hindered adoption of EBVs by smaller breeding programs, and programs without access to the required expertise. To address this need, IWDR includes EBV calculation and data management tools that are accessible to all dog breeders. Through the IWDR database, breeders can obtain EBVs, allowing them to objectively identify which young dogs are most likely to produce puppies that move the population's average phenotypic merit closer to the breeding goals defined by the breeding manager.

## Six Step Approach to EBV Selection

**Figure 2:**
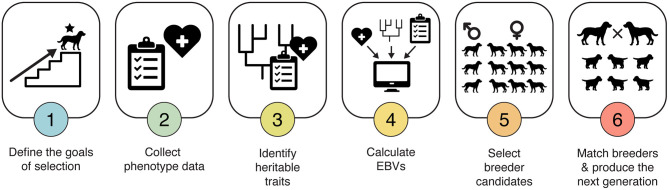
Six-step approach to EBV selection. Implementing an EBV-based selection program can be daunting. By distilling it down to just six steps, each supported by tools and training available through IWDR, this method of making breeding decisions can be made accessible to both working dog breeders and hobby breeders.

Implementing an EBV-based selection program can be daunting. Here, we distill the process into six steps (Figure 2). Using this approach, breeding programs can systematically apply the scientific principles of population genetics and genetic selection to their dog populations.

### Step 1. Define the Goal(s) of Selection

Before applying any selection in a breeding program, it is critical to establish clear goals, such as improving success rates. With the goals defined, the program can then identify measurable phenotypic traits relevant to achieving those goals.

### Step 2. Collect Phenotype Data

A protocol must be developed to uniquely identify each dog and to uniformly and accurately measure each trait of interest on all breeding dogs and on all or most of their progeny over successive generations. For behavioral traits, this might be the Behavior CheckList (35), while for a trait like hip dysplasia, the PennHIP (36, 37) or extended view radiograph (OFA, BVA, FCI) scores could be used (38). All data should be stored in a secure, uniformly coded, electronic database like IWDR.

### Step 3. Identify Heritable Traits

Using the phenotype data and pedigrees stored in the database, the ***heritability*** of each phenotypic trait measured in step 2 must be calculated. The more heritable a trait is, the bigger the response will be to one generation of selection (39). Traits with a heritability of at least 15% are considered good candidates for genetic selection. With accumulating knowledge derived from genomic information, genetic improvement in traits with even lower heritability may eventually be feasible.

### Step 4. Calculate EBVs

For the traits selected in Step 3, EBVs can be calculated using specialized software that combines phenotype data with the pedigree structure (25, 40, 41). Models fitted with this software can accurately account for overlap in the genetic background influencing different traits and for external, non-genetic factors that produce variation in phenotypes, such as the season of year or age of the dog when the phenotype was measured. Access to EBVs calculated using this software is available through IWDR.

### Step 5. Select Breeder Candidates

Young dogs with high genetic merit from the EBV analysis in Step 4 should be evaluated in detail to assess their suitability as breeders, with a focus on the whole dog. Metrics considered will typically include suitable conformation, reproductive capacity, health screening typical for the breed and a performance assessment.

### Step 6. Match Breeders and Produce the Next Generation

From among the candidate dogs identified in Step 5, mating pairs are chosen, such that weaknesses in one dog are complemented by strengths in the mate. In addition, it is important to choose pairs that maximize the genetic diversity of the breeder pool, minimize inbreeding in litters, and limit the number of progeny any single individual produces in their lifetime (31). A practical guideline is to attempt to limit the average increase in the **coefficient of inbreeding** to no more than 2% per generation.

To achieve this goal, an easily implemented strategy is to limit the number of litters produced by any single parent (42). While inbreeding could be kept to a minimum by allowing each parent to produce only a single litter, this isn't operationally feasible, and thus tradeoffs must be made. One practical solution is to restrict each male to siring no more than 8 litters and each female to producing no more than 3 or 4 litters. In a population producing ~200 puppies per year, this will limit the rate of inbreeding increase to no more than 2% per generation. We note that, as the rate of inbreeding increase is related to effective population size (39), chapter 4, p. 65], any workable strategy for maximizing the effective population size will limit inbreeding.

## Implementing an EBV Selection Program

While the six steps described above provide a high-level perspective on EBV selection, the reality of starting such a program can be daunting. Here, we describe how Guiding Eyes for the Blind (Guiding Eyes, hereafter), implemented their EBV selection program, and highlight some of the challenges they needed to overcome. Guiding Eyes have shared their breeding program data to illustrate this process (43). Their experience illustrates the dynamic nature of any breeding program. The outcome of each of the six steps is not fixed, and often must be revisited and revised based on information acquired as the process evolves over time.

The Guiding Eyes for the Blind breeding colony collectively produces about 520 weaned puppies (about 90% Labrador retrievers and 10% German shepherds) each year. Dogs who do not succeed as guide dogs are either moved to other organizations or adopted out to pet homes, depending on their testing results (44). Currently, Guiding Eyes has 120 active Labrador retriever breeders (82 female and 38 male) and 13 active German shepherd breeders (10 female and 3 male), as well as frozen semen from 20 Labrador retrievers and 13 German shepherds with high selection indexes.

Guiding Eyes started using genetic selection with EBVs in their Labrador retrievers in 2003. Their German shepherd colony is too small to calculate EBVs, but Guiding Eyes is working with IWDR to address this through collaboration and data sharing with other guide dog breeding colonies.

### Implementing Step 1. Define the Goal of Selection

Guiding Eyes made the decision to move toward implementing EBV selection in 1995 with the goal of improving health and behavior traits, while preserving genetic diversity. At that early stage, they did not have the data infrastructure needed to identify which traits were most correlated with those outcomes, and thus might be the targets of selection. The collection of phenotype data (Step 2) was critical in making this determination.

### Implementing Step 2. Collect Phenotype Data

Guiding Eyes first had to set up a data management system (a relational database) for collecting phenotype, health, and pedigree information, and populate it with information on their dogs. Using this database, Guiding Eyes identified hip quality, elbow quality, soft trachea and allergic/atopic dermatitis as top health reasons for dogs failing out of the program. Although nearly twice as many dogs failed for temperament or behavioral reasons, rather than health reasons, Guiding Eyes did not initially have a useful system for scoring non-health traits. Behavioral trait data was being collected using the Canine Behavioral Assessment and Research Questionnaire (C-BARQ), but the estimates of heritability for C-BARQ measured traits were very low, and insufficient for driving genetic improvement (45).

**Figure 3:**
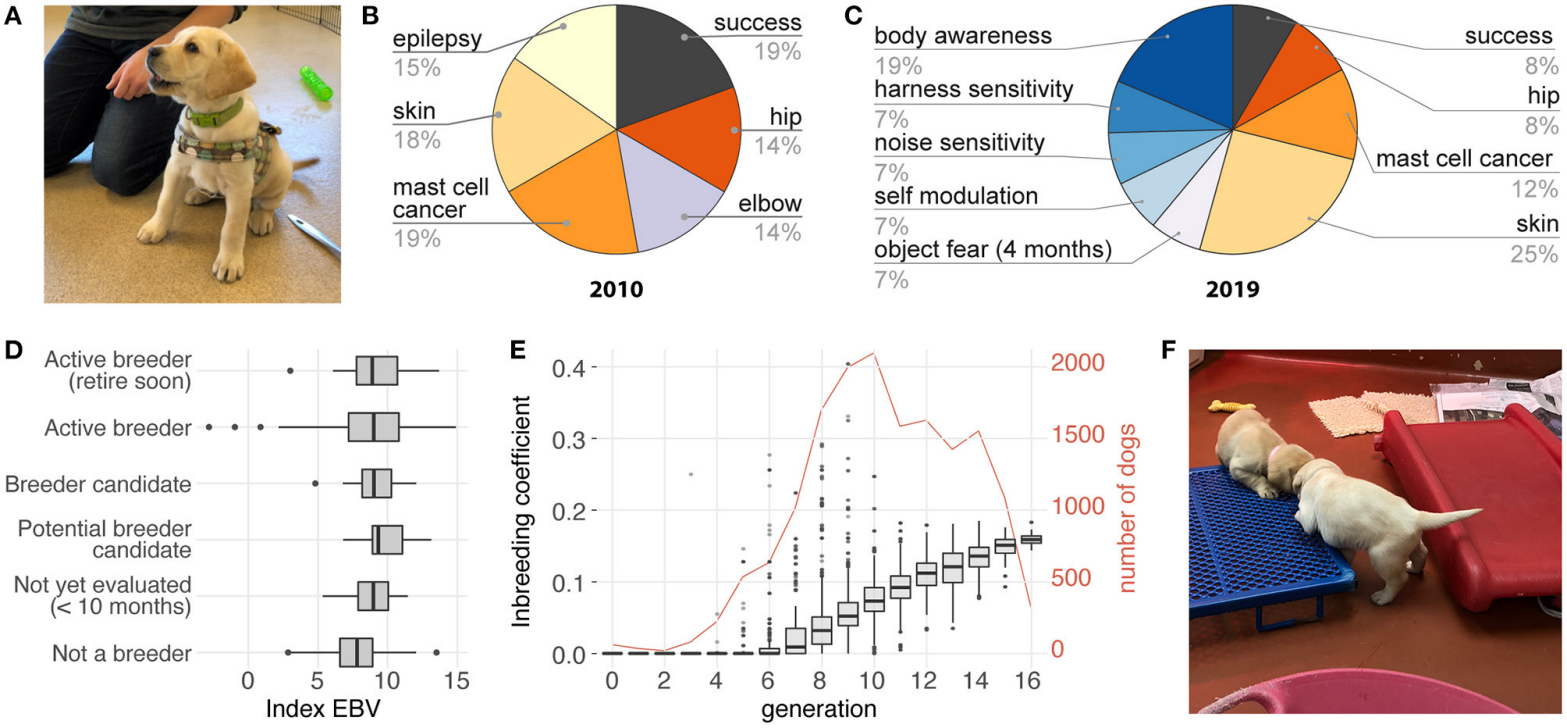
Implementing EBV selection at Guiding Eyes for the Blind. **(A)** The behavioral phenotypes are collected using the Behavior CheckList scored by trained observers during standardized tests and assessments starting at 2 months of age, and continuing through training until about 26 months of age. **(B)** The selection index, which weights each trait based on its importance to the breeding program, has changed over time to include more behavioral phenotypes. **(C)** The current index includes about 50% behavioral and 50% health traits. **(D)** The rate of genetic improvement can be monitored by reviewing the selection index scores for the current population of dogs. Here, dogs not used for breeding have, on average, the lowest scores, while active breeders, including those nearing retirement, have the highest scores, consistent with the increasing genetic improvement expected in a successful breeding program. If genetic improvement plateaus or worsens, the genetic model, data quality, change in testing protocols or criteria, weighting in the index and other influences are reviewed and corrective action taken. **(E)** Inbreeding is minimized by selecting mate pairs that maximize diversity, and by limiting the number of progeny produced by any single individual. Between generation 8 and 16, average inbreeding coefficient increased from 3.4 to 16%, corresponding to an increase of 1.6% per generation. **(F)** To produce high-performing working dogs, environmental factors that influence success must also be addressed. At Guiding Eyes for the Blind, for example, kennels with nursing puppies are enriched with different surface textures, noises, novel objects, and other experiences the puppies will need to be familiar with as working dogs. Image credits **(A,F)**: Elinor K. Karlsson.

It took a global collaboration of working dog breeders 10 years to develop a tool for assessing behavioral traits with the standardized terminology, inter-rater reliability, and score resolution needed for genetic selection. The Behavior CheckList, which is the tool recommended by IWDR, is an optimized version of the scoring tool originally used to validate construct validity of the C-BARQ, and incorporates measures known to affect guide dog performance, including noise sensitivity, harness sensitivity, and body sensitivity (Figure 3A; Supplementary Material) (45). Behavior CheckList scores are assigned by trained personnel with experience in behavioral coding. At Guiding Eyes, behavioral coding has been correlated with behavioral information captured by ECG, accelerometry and gyroscope data on ~2 month old puppies (46).

The Behavior CheckList was originally designed for assistance and guide dogs, but it may be generalizable to other types of working dogs, such as detection dogs, when augmented with additional job-specific phenotypes (e.g., hunt drive, indication, bite) (47).

### Implementing Step 3. Identify Heritable Traits

Traits included in an EBV selection program need to be both relevant to the high-level goals of the program, and sufficiently heritable to respond to selection. For each trait measured in the Guiding Eyes population, and collected in the database, the heritability was estimated using statistical models that considered sex, age, weight, and other features when appropriate (Table 1).

**Table 1:** Heritability and genetic selection at Guiding Eyes.

**Trait**	**Estimate of** **heritability (%)**	**No index**		**Weighting in index**				**Software used**
		**2003**	**2005**	**2010**	**2014**	**2016**	**2019**	
Success	46			20%	23%	35%	8%	R
Health	44			–	–	–	–	R
**Health-related measurements**								
Elbow quality	63	EBV	EBV	14%	–	–	–	R
Epilepsy	62			15%	12%	8%		R
Soft trachea (STEBV)	61			–	–	–	–	R
Tricuspid valve dysplasia (TVD)	56			–	–	–	–	R
Hip quality	52	EBV	EBV	14%	–	–	8%	R
Skin (Allergic/atopic dermatitis, otitis, etc.)	39		EBV	18%	21%	21%	25%	BLUPF90
Mast cell cancer (age of onset)	28			19%	11%	14%	12%	R
**Behavior-related measurements**								
Activated by stress (puppy test)	56			–	–	–	–	BLUPF90
Noise sensitivity (puppy test)	54			–	–	–	7%	BLUPF90
Thunderstorm phobia	50			–	15%	–	–	R
Inhibited by stress (puppy test)	48			–	–	–	–	BLUPF90
Harness sensitivity (2-trait with IFT EBV)	47			–	18%	10%	7%	BLUPF90
Body awareness (3-trait with IFT EBV)	36			–	–	12%	19%	BLUPF90
Self-modulation (puppy test)	34			–	–	–	7%	BLUPF90
Object fear (puppy test)	32			–	–	–	7%	BLUPF90

*Genetic selection with EBVs will be most effective on traits that have a heritability > ~15%. Heritability measures are specific to a particular population and a particular environment, requiring them to be estimated for each breeding population. Here, we show heritability estimates for the Guiding Eyes for the Blind Labrador retriever population, which range from a low of 28% (mast cell tumor) to a high of 63% (elbow quality). “Puppy test” indicates testing done at ~2 months of age, and IFT (In For Training) indicates testing done at ~16 months of age. We also show how traits were weighted in the overall selection index, and how those weightings change over time. “EBV” indicates traits for which EBVs were calculated prior to the development of the selection index. A dash indicates a trait not included in the selection index*.

### Implementing Step 4. Calculate EBVs

Guiding Eyes started calculating EBVs in 2003 for two traits: elbow quality and hip quality (Table 1). EBVs were estimated using MTDFREML (MTGSAM for binary threshold traits) (48, 49). By 2010, they had collected sufficient data to develop their first overall selection index, combining and weighting EBVs for five health traits, selected because they were most responsible for dogs failing prior to beginning training, and a binary measure of training success (Figure 3B). The emphasis (relative weight) placed on each health trait was proportional to the percent of rejections caused by that trait. The standardized weight was calculated by dividing the relative weight by the genetic standard deviation of the trait. To calculate the overall selection index value for each dog, the EBV for each trait was multiplied by the standardized weight for that trait, and the weighted EBVs for all traits then added together.

Selection by EBVs is an inherently dynamic process, because the selection itself changes the frequency of traits in the population, so the selection index is reviewed and revised regularly to focus on traits of highest priority in the current population (Table 1). By 2014, Guiding Eyes had sufficient Behavior CheckList data to incorporate EBVs for behavioral phenotypes, starting with thunderstorm phobia and harness sensitivity. Before adding a new behavioral EBVs into the overall index, its utility is assessed by someone knowledgeable about the colony, to confirm that dogs are ranked generally as expected. This less quantitative “sniff test” is important for affirming the real world relevance of the EBV.

Today, behavioral and health traits are equally represented in the Guiding Eyes selection index (Figure 3C). Each dog's score on the selection index is the primary tool used to decide which young dogs should be considered as breeders (step 5). EBVs, and the overall selection index score for each dog, are recalculated every 2 weeks to coincide with the selection of new breeder candidates, ensuring that all phenotype data are included in the calculations.

### Implementing Step 5. Select Breeder Candidates

Typically, Guiding Eyes replaces about one third of its breeding colony each year. An increase in average genetic merit means that animals in the next generation will have more desirable genetic potential than their parents (Figure 3D). While Guiding Eyes prioritizes dogs with a high selection index values as breeder candidates, the decision to keep a dog for breeding is not based on those scores alone. Each month, 9 females and 3 males (usually 15–17 months old) are selected as breeding candidates and undergo additional behavioral, health and reproductive screenings. Dogs may be excluded based on weight, heritable conditions not captured in the selection index, reproductive abnormalities (e.g., cryptorchidism), primary persistent anestrus or abnormal estrus patterns, or undesirable conformation. In addition, they consider the individual dog's longitudinal behavioral scores and behavioral trends in the litter. Finally, they consider how closely related the candidate is to other dogs in the breeding colony, favoring the less related dogs.

### Implementing Step 6. Match Breeders and Produce the Next Generation

When Guiding Eyes matches dogs for breeding, it tries to balance weaknesses in one dog with strengths in the mate. It assesses each potential pairing using three primary criteria: (1) the dogs have not been previously bred together; (2) their progeny would have a lower predicted coefficient of inbreeding compared to pairings with other dogs; (3) neither dog is affected by a genetic disease caused by a known mutation, and, at most, only one of the pair is a carrier. In order to avoid popular sire effects, Guiding Eyes tries to use all stud dogs equally.

## Genetic Change Due to Selection in the Guiding Eyes Breeding Program

**Figure 4:**
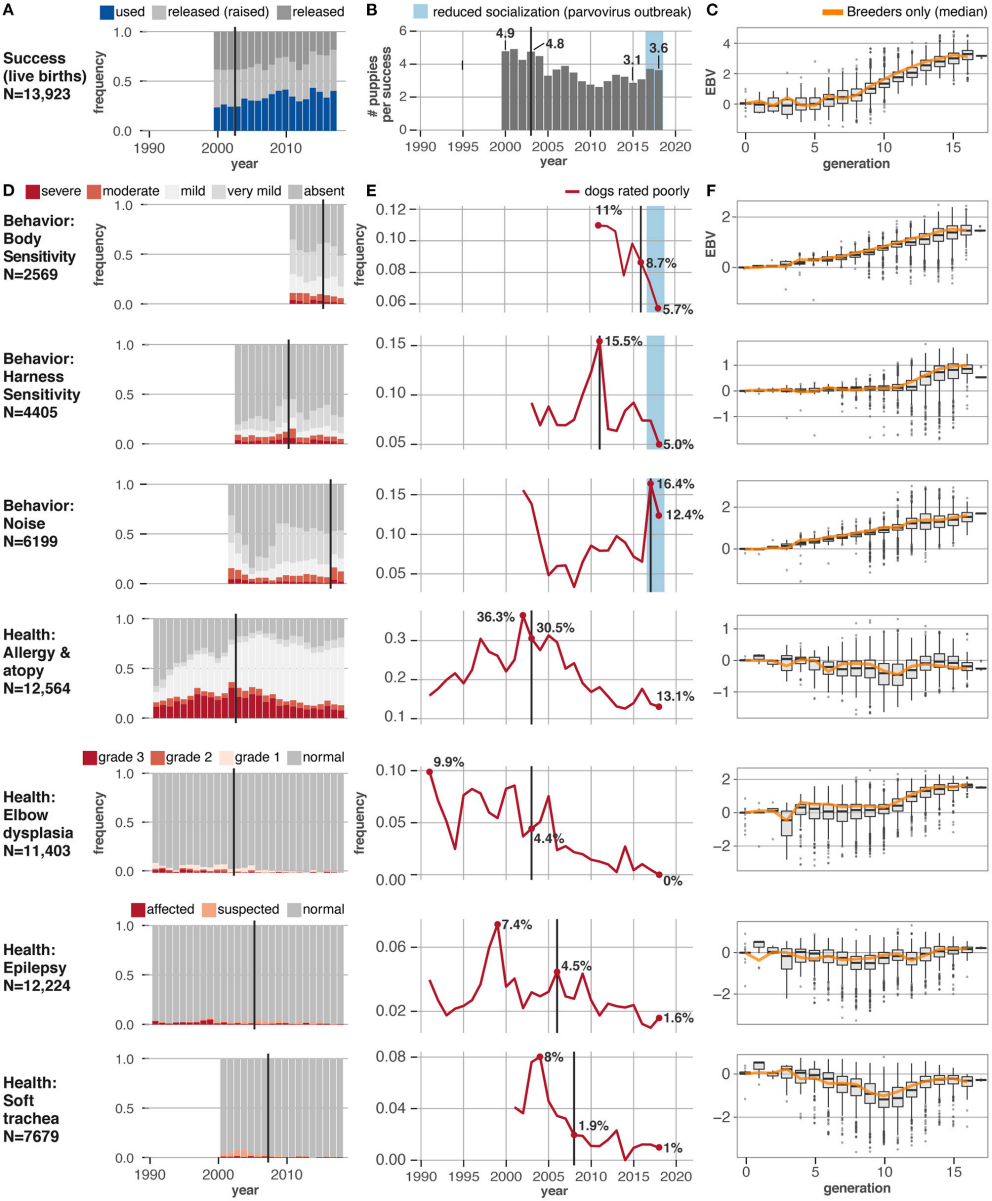
Behavioral and health traits change in response to EBV selection. Eight traits under selection in the Guiding Eyes for the Blind population show varying responses to the onset of selection using EBVs. Vertical black lines show when EBV selection for each trait was started. **(A)** Change in fraction of dogs successfully placed as guide dogs per year, starting in 2000, when the current assessment criteria were implemented. **(B)** With improving success rates, the number of puppies produced by Guiding Eyes for each successful dog has dropped over time. **(C)** The EBV scores for success, as a trait, have increased over the last 17 generations of selection, indicating an improvement in genetic merit with each successive generation, even if just the animals selected as breeders are considered (orange line). **(D)** Change in relative frequency per year of different phenotypes, starting with the year the phenotype data started being collected. **(E)** Change in the fraction of dogs with a disqualifying phenotype (red lines). **(F)** The EBV scores for each trait over the last 17 generations of selection show increasing EBVs, indicating an improvement in genetic merit with each successive generation. A similar trend in the median EBV scores is seen if just the animals selected as breeders are considered (orange line).

Overall, the breeding program at Guiding Eyes has been very effective, illustrating the power of EBV selection. Most importantly, Guiding Eyes was able to reduce the number of puppies born each year, from 4.8 to 3.6 pups born, for each successful dog (Figures 4A–C). Given the enormous resources required to train even a single dog, this achievement has allowed Guiding Eyes' to more efficiently fulfill its mission of providing high-performing dogs to people with vision loss.

Guiding Eyes saw an improvement in the EBVs for every trait included in the selection index (Figures 4C,F). Elbow quality, and scores for behavioral traits, including harness and body sensitivity, and noise sensitivity, all improved (Figures 4D,E) The incidence of allergic/atopic dermatitis, idiopathic epilepsy and soft trachea all dropped. When using phenotype based selection, the number of dogs diagnosed with allergic/atopic dermatitis, elbow dysplasia, and idiopathic epilepsy had varied widely from year to year, but once EBVs were employed, a consistent genetic improvement was evident. Throughout the breeding program, inbreeding coefficients slowly increased at an average rate of 1.6% per generation (Figure 3E).

Several interesting features are evident in the data. First, environment matters (Figure 3F). In 2017 there is a sharp rise in dogs exhibiting moderate and severe noise fear (Figure 4E). This coincides with an outbreak of parvovirus in the breeding facility, which shut down the early socialization program for puppies. This program was designed specifically to expose dogs to a wide range of stimuli, including different noises, during a critical period of development, and its curtailment was detrimental.

Second, how traits are defined for the selection index can lead to unexpected consequences. Between 2010 and 2011, before the Behavior CheckList was in use, the only behavioral metric used in the selection index was a binary trait reflecting whether or not a dog successfully completed the guide dog training program. Selecting for this trait inadvertently increased the incidence of excessive body sensitivity in the population. A closer look revealed that using success as a metric favored dogs who were easy to control, and many of these dogs had heightened body sensitivity. Once the Behavior CheckList measurements of body sensitivity were incorporated into the selection index, the increasing trend in body sensitivity was reversed.

## Implementing EBV Selection With IWDR

While the experiences of Guiding Eyes illustrate the potential of EBV selection and how to effectively implement this six-step approach, EBVs have not been widely adopted by working dog breeding organizations because most programs, on their own, have neither the dog population size nor the staff expertise to implement it successfully. To make EBV selection accessible to all working dog breeding programs, regardless of the size of their breeding colony or the expertise of their staff, a different approach is needed. IWDR supports both working dog and hobby breeders of any breed interested in using EBVs to achieve genetic improvement. It offers state-of-the-art tools, expertise, and training that, until now, were accessible to only the largest canine breeding colonies.

### Database

For breeding programs, establishing and maintaining a database, and managing data storage, is costly and difficult. IWDR provides a secure, uniformly coded, electronic database accessible through a paid subscription (International working dog registry—dogs serving humanity^1^) from anywhere in the world. IWDR facilitates storing phenotypes, pedigrees, and genetic data.

### Phenotyping

Many breeding programs still use non-standardized phenotype scoring approaches that are not ideal for EBV selection. IWDR provides expert training and resources for standardized phenotype scoring, including for behavioral traits via the Behavior CheckList (35).

### Tools to Estimate Heritabilities and Calculate EBVs

Breeding programs are often too small to use EBV selection, or lack the required expertise. IWDR stores phenotypes using a standardized codebook, making it possible to pool data from many organizations. Using pooled data, IWDR can estimate heritability and calculate EBVs using all dogs of a specific breed, while keeping the detailed data for each organization secure and private. EBVs calculated by IWDR will be far more accurate than those from a single, small breeding program, because they are based on many more observations, thus enabling all breeding programs, regardless of colony size, to utilize EBVs. With these values, breeding managers can assess which are the genetically most desirable dogs within their colony to keep for breeding, and they can compare their organization's EBVs to the population average, thus quantifying where their dogs rank relative to all dogs of that breed in the database.

To calculate EBVs, IWDR uses the BLUPF90 suite of programs (40, 41). Most health and behavior phenotypes are categorized into five classes, ranging from one (least desirable) to five (most desirable), and are modeled as linear mixed models, with fixed effects included to adjust for the presence of environmental effects known to produce phenotypic variation. IWDR can also calculate EBVs for binary traits by analyzing them as threshold traits using the Gibbs sampling methodology implemented in the BLUPF90 program suite. Once EBVs are calculated and stored into IWDR, they are presented with both an assessment of accuracy and each dog's percentile ranking amongst all dogs from that breed in the database.

### Pairing Dogs for Breeding

Selecting dogs for breeding is a complicated task, as EBV scores, predicted inbreeding, and health are all considered. IWDR provides an easy-to-use tool that calculates the predicted inbreeding for a potential litter before the mating is actually done, and summarizes both the phenotype data and the genetic test data available on possible mates. IWDR can also compare EBVs between breeding programs, enabling the exchange of breeding stock with less risk of damaging short term outcomes for dogs. This is critical for balancing selection goals with breeding for improved genetic diversity and population health.

### Integrating Genomic Relatedness

Genomic data from either marker genotyping or whole genome sequencing can more precisely define the exact degree of genetic relatedness that exists among pairs of dogs than a pedigree alone (50). IWDR is developing tools that will incorporate the use of genomic data into the EBV calculations, thus enabling breeders to work with EBVs estimated with higher accuracy.

## Building the Next Generation of Genomic Tools

Today, it is not possible in dogs to accurately predict health or behavioral traits from genome sequence data alone. For a small number of single-gene diseases, genetic tests are available, allowing carriers of disease risk factors to be identified [although with some important caveats (51)]. Single-gene diseases, however, are just a tiny fraction of heritable diseases, and don't include cancer and other common diseases that are the major causes of death in dogs (52).

The vast majority of diseases, and behavioral traits, are genetically complex, shaped by changes in hundreds, and even thousands of genes, as well as non-heritable environmental factors (53–55). For these traits and diseases, applying selection is difficult without using some aids to improve the accuracy of selection decisions. Active selection of desired genetic variation can lead to unintentional accumulation of deleterious genetic variation due to “hitchhiking” of deleterious alleles physically linked in the genome (56), and unintentional, undesired effects if the genetic variants under selection are pleiotropic, or have effects on multiple traits. Without understanding the genetic basis underlying desired or undesired phenotypes, artificial selection for specific traits in domestic dog breeds can increase deleterious genetic variation (57).

The Working Dog Project and IWDR are working together to build the next generation of genomic tools for dogs, in partnership with dog breeders willing to share de-identified genomic data and phenotypes from their dogs. Genomic technologies have the potential to substantially improve selective breeding in dogs, but it will require extremely large datasets to develop robust, high-quality tools. By working together, and pooling data and expertise, we can develop two new approaches to dog breeding that could dramatically improve the supply of high-quality, healthy working dogs: ***genomically enhanced*** ***EBVs (sometimes abbreviated to gEBVs)*** and ***genomic breeding values (genomic*** ***EBVs)***.

### Genomically Enhanced EBVs

EBVs that incorporate genomic data are known as genomically enhanced EBVs. Compared to traditionally calculated EBVs, genomically enhanced EBVs can increase accuracy of genetic merit predictions by as much as two-fold, because genomic information complements pedigree information (27). In practical terms, this means that the accuracy of well-estimated genomically enhanced EBVs for young dogs can be, in some situations, as accurate as if those dogs had already produced 20 progeny who have already matured to acquire their own phenotypes. Compared to EBV selection, genomically enhanced EBV selection is more effective, especially for traits with low heritability (58, 59).

Using genomically enhanced EBVs can make dog breeding programs more efficient. Generating high accuracy EBVs (>0.80) requires phenotypes for both parents and their progeny, a challenge for late age onset conditions, like cancer and epilepsy. The addition of DNA marker information in the form of genomically enhanced EBVs should increase the accuracy of genetic merit prediction in young puppies, thus enabling more accurate selection decisions to be made before they produce progeny. This reduces the generation interval and costs associated with waiting for animals to mature. In addition, a DNA sample collected from a young puppy could identify, at a very young age, dogs that should not be further developed for a particular line of work, but rather should become beloved pets, avoiding costly care and training.

### Genomic Breeding Values

A genomic EBV is a prediction of a dog's relative genetic merit based solely on its genome sequence. No pedigree information is required. With genomic EBVs, a decision could be made to keep a young dog for breeding as soon as a DNA sample can be collected, a method known as ***genomic selection*** (60). Selecting animals based on their genome sequence, rather than waiting to measure phenotypes later in life, can accelerate genetic gain by reducing the generation interval.

While genomic selection offers promise for assessing relative genetic merit among young puppies, literally thousands of whole genome sequenced dogs with their well-measured phenotypes will be needed in one dataset in order to develop the prediction equations (61, 62). Once developed for a particular breed, these prediction equations should enable even the prediction of relative genetic merit of dogs from outside breeding programs or dogs living in shelter populations (63, 64). If sufficiently large datasets of full genome sequences and phenotypes from many breeds can be assembled, prediction equations that work in any dog, regardless of breed, may be feasible (60).

### Very Large Sample Sizes Are Essential

The key to developing genomically enhanced EBVs and genomic breeding value technology for working dogs will be assembling very large datasets. Both approaches require studies with tens, or even hundreds, of thousands of dogs, each with phenotype information for traits of interest (60, 65), and coordination between working dog breeders, statistical geneticists, and data scientists. The partnership between IWDR and the Working Dog Project is designed to address this challenge. IWDR is a platform for obtaining both whole genome sequences and uniformly coded phenotypes for tens of thousands of dogs. With consent from the dog breeder, these data, once de-identified, can be shared with scientists with expertise in complex trait mapping and statistical genetics through the Working Dog Project. All data contributed to the Working Dog Project will be part of an open data repository, encouraging even scientists unfamiliar with dog breeding to contribute their skills. No breeding program, on its own, has either enough dogs or the expertise to solve this challenge, but by pooling resources, all working dog breeders can benefit.

## Conclusion

Large-scale genomic studies, such as those we propose for working dogs, have much broader ramifications outside of improved breeding practices. Genomically enhanced EBVs, for example, can predict which dogs are likely to be high-performing working dogs before the investment of significant resources into their training. With big enough datasets, we'll be able to make these predictions in any dog, including shelter dogs, some of whom might be more suited to a high-energy working dog career than life as a pet—thereby opening up new sources for these high-demand dogs.

Ultra-large-scale dog genomics can also support advances in veterinary medicine. In human medicine, genomically enhanced EBVs are known as polygenic risk scores (23), and are already used to predict risk of heart disease at a young age, when risk reduction through environmental changes is still feasible (66). Similarly, given enough data, we should be able to develop risk scores for complex diseases like cancer and heart disease for veterinary medicine, identifying which dogs should be most carefully monitored, or when interventions should be more aggressive.

Finally, large-scale genomics is the first step in discovering the fundamental genetic basis for health and behavioral traits (67, 68). Aging and disease in dogs show strong similarities to humans, and finding the cellular mechanisms responsible in dogs could provide new clues for treating humans as well (69), ultimately resulting in better diagnostics, therapeutics, and lifestyle interventions for dogs and humans alike.

For complex traits like behavior, which reflect the interaction of genes with the environment, predictions based on genomic data alone will never be perfect. Environment, especially early in life, has a profound effect on the development of the brain. If a mother is not able to perform intrinsic nesting behaviors, or escape from her pups for a while, this stress can lead to aberrant working behaviors in her pups (70). While still in the breeding kennels, working dog puppies develop vision, fear, social behavior, and cognitive abilities, and how these characteristics develop reflects environmental cues. A breeding program that does not provide positive early exposure to a variety of sounds, scents, textures, and visual stimuli will leave their dogs more fearful of the world around them, and less able to perform their job. On the other hand, breeding programs that do provide puppies with a wide range of novel, and positive, experiences in a developmentally appropriate way will maximize the return on an investment in improved breeding practices.

Collaboration and data sharing are essential if we hope to improve the supply of high performing working dogs. Sharing the details, and outcomes, of ongoing breeding programs, including both successes and failures, will help develop better genetic selection techniques for dogs. Furthermore, no single organization can hope to reach the massive sample sizes required to develop the next generation of genomic tools. When searching for the genetic causes of a complex trait, the bigger, the better, and there is no clear upper bound on how many samples are enough (65). By working together, IWDR and the Working Dog Project will promote innovation and collaboration via advanced genomic technologies and open science, and provide the practical tools dog breeders need to implement these advances. This collaborative strategy is the only practical path for realizing the potential of genomics to transform working dog breeding, and to address health challenges in both dogs and humans.

## Author Contributions

EK, EL, and FLC equally contributed to the formation of the manuscript's conceptual ideas and framework. All authors contributed to writing and editing the manuscript.

## Conflict of Interest

EL was contracted by IWDBA for IWDR development. FLC was employed by the company Cellular Longevity, Inc. dba Loyal. The remaining authors declare that the research was conducted in the absence of any commercial or financial relationships that could be construed as a potential conflict of interest.

## Publisher's Note

All claims expressed in this article are solely those of the authors and do not necessarily represent those of their affiliated organizations, or those of the publisher, the editors and the reviewers. Any product that may be evaluated in this article, or claim that may be made by its manufacturer, is not guaranteed or endorsed by the publisher.

## Footnotes

^1^Available online at: http://www.iwdr.org (accessed May 31, 2021).

## Glossary

Coefficient of inbreeding: A measure, ranging between 0 and 100%, of the degree of homozygosity present in the genome of an individual. Animals with low (<10%) or no inbreeding are more genetically diverse than individuals with higher inbreeding coefficients. Animals that are 100% inbred are genetically identical, to the point that tissue like skin grafts transferred from one to another are readily accepted by the body.
Complex trait (or complex disease): Any trait whose inheritance cannot be explained by variation in a single gene. Complex traits (like behavior traits) and diseases (like cancer) are polygenic, shaped by variation in hundreds of genes, and by the environment, and tend to show a continuous range of variation.
Estimated breeding value (EBV): Statistical measure of relative genetic merit of an individual compared to others in the population for a given heritable trait. To obtain an EBV for each member of a breeding population requires collecting pedigrees and an accurately measured trait on at least several hundred or more genetically related animals.
Genetic merit: Animal breeding term describing how an animal ranks relative to other individuals being considered as prospective parents of the next generation of offspring in a selective breeding program.
Genomically enhanced EBV (gEBV): Produced by incorporating genomic (DNA-based) information into the statistical model that generates an EBV; either by improving estimates of relatedness using genomic data and/or by associating genomic data with variations in the desired trait.
Genomic breeding value (genomic EBV): Measure of genetic merit of an individual relative to others in the population using only DNA based information.
Genomic selection: Selection that uses DNA based information; in animal breeding this specifically refers to methods using genomic data (such as SNP markers or whole genome sequencing data) associated with specific trait(s) to directly estimate the genetic merit without first observing the trait in that animal.
Genotype: The genetic complement of an individual inherited from its parents. This term is also used to describe the pair of alleles observed at a single locus in an individual.
Phenotype: The observable characteristics, or traits, of an individual that result from the interaction of genetics with environment. A single phenotype can reflect the influence of many different genes (see complex trait).
Phenotypic selection: Traditional method of selecting which individuals breed based on whether or not they display desired traits.
Polygenic risk scores: a measure of the likelihood that a person's genetic makeup will eventually lead to expression of a particular disease or human health anomaly. Polygenic risk scores are widely used in human medicine and human genetics. The underlying statistical methods for calculating these scores are mathematically equivalent to the methods used for calculating estimated breeding values or genomically enhanced breeding values.
Postzygotic selection: Selection that occurs after fertilization (for example, by providing some adult animals with extra food resources). In contrast, prezygotic selection includes deciding which individuals will mate, and is common in modern dog breeding.
Selection index: Method for determining an individual's overall genetic merit. It is best used for making selection decisions with respect to multiple traits at once by weighting each trait based on its importance to the breeding program.
Heritability: The percent of total variation observed in a trait within a population that is genetically transmitted from parents to their offspring. Heritability can range from 0 to 100%. If a trait is highly heritable, like coat color, it suggests a dog's phenotype is determined largely by genetic factors. Heritability measures are specific to a particular population and a particular environment.
International Working Dog Registry (IWDR): A nonprofit resource consisting of a secure online database storing uniformly coded phenotypic and genotypic information on working dogs. IWDR was formed by experts in the working dog breeding field whose goal is to provide data driven genetic selection tools for dog breeders. Website: www.iwdr.org.
Working Dog Project: Part of the non-profit Darwin's Ark Foundation (DarwinsArk.org), the Working Dog Project promotes ultra-large-scale dog genetic research following an “open-science” model. This approach will advance our understanding of complex behavioral traits and diseases in all dogs, and enable the development of powerful genomic tools for working dog breeders. Website: https://workingdogproject.org/.
