# Supplementary material for: Advancing Genetic Selection and Behavioral Genomics of Working Dogs Through Collaborative Science
Source: Front Vet Sci. 2021 Sep 6;8:662429. doi: 10.3389/fvets.2021.662429 (PMC8450581; doi:10.3389/fvets.2021.662429)
Supplement: Supplementary file 1 [file Data_Sheet_1.pdf]

Behavior Checklist

Dog Name: ____________

Dog ID: ____________ **Type of Evaluation:** ____________

Date: ____________ **Evaluator:** ____________

1. **ANXIOUS IN UNFAMILIAR LOCATIONS -** Initially anxious during first visits to unfamiliar locations. Exhibited as increased or inhibited activity, cautious and/or less responsive or focused

☐ Abs ☐ VMild ☐ Mild ☐ Mod. ☐ Sev. ☐ N/A ☐ RFR

Comments:

2. **NOISE SENSITIVITY** - Startle, tense body language, hurries, escape, or displacement behaviors when exposed to noise such as car horns, hair dryers, vacuums, banging

☐ Abs ☐ VMild ☐ Mild ☐ Mod. ☐ Sev. ☐ N/A ☐ RFR

Comments:

3. **FEAR OF NOVEL OBJECTS -** Fearful, avoidant or suspicious of unfamiliar objects, which could be anything, but common examples are plastic bags, statues, yard equipment, balloons, etc.

☐ Abs ☐ VMild ☐ Mild ☐ Mod. ☐ Sev. ☐ N/A ☐ RFR

Comments:

4. **FEAR OF UNDERFOOTINGS** - Fearful, nervous, apprehensive of various walking surfaces

☐ Slippery Floors ☐ Gratings ☐ Other

☐ Abs ☐ VMild ☐ Mild ☐ Mod. ☐ Sev. ☐ N/A ☐ RFR

Comments:

5. **FEAR OF DOGS -** Inhibited response to other dogs on leash or off leash such as low body posture, pulling back, head low, apprehensive, avoidant, hackles, withdraws, reluctant to proceed, watches

☐ Abs ☐ VMild ☐ Mild ☐ Mod. ☐ Sev. ☐ N/A ☐ RFR

Comments:

6. **FEAR OF STAIRS** - Hurries, refuses, or hesitant on stairs

☐ open back stairs ☐ open grate stairs ☐ up stairs ☐ down stairs ☐ closed stairs

☐ Abs ☐ VMild ☐ Mild ☐ Mod. ☐ Sev. ☐ N/A ☐ RFR

Comments:

7. **FEAR OF TRAFFIC -** Tense body language, tail low or tucked, change in activity level, startle, retreat, escape in response to sight and sounds of vehicle traffic

☐ Abs ☐ VMild ☐ Mild ☐ Mod. ☐ Sev. ☐ N/A ☐ RFR

Comments:

8. **SEPARATION ANXIETY -** Restless, vocalizes, and /or becomes destructive when left alone

☐ in crate

☐ Abs ☐ VMild ☐ Mild ☐ Mod. ☐ Sev. ☐ N/A ☐ RFR

Comments:

9. **HYPER -ATTACHMENT -** Overly attached to primary handler, seeks to return to primary handler when handled by others

☐ Abs ☐ VMild ☐ Mild ☐ Mod. ☐ Sev. ☐ N/A ☐ RFR

10. **FEAR OF STRANGERS -** Fearful, nervous, apprehensive with strangers, cautious, avoidant, hackling, slow approach, keying in, barking, growling, escape

☐ Abs ☐ VMild ☐ Mild ☐ Mod. ☐ Sev. ☐ N/A ☐ RFR

Comments:

11. **BODY HANDLING CONCERN** - Avoidant, anxious, fearful and/or aggressive when handled for non-invasive activities in a manner typical of a vet exam, grooming and/or nail clipping

☐ Nails ☐ Restraint

☐ Abs ☐ VMild ☐ Mild ☐ Mod. ☐ Sev. ☐ N/A ☐ RFR

Comments:

12. **RETREATS WHEN REACHED FOR -** Moves head or face away when reached for by familiar persons

☐ Abs ☐ VMild ☐ Mild ☐ Mod. ☐ Sev. ☐ N/A ☐ RFR

Comments:

13. **HARNESS SENSITIVITY -** Drops rear quarters when harness handle lays on back.

☐ Abs ☐ VMild ☐ Mild ☐ Mod. ☐ Sev. ☐ N/A ☐ RFR

14. **AVOIDANCE OF BLOWING FAN -** Fearful or avoidant when walking past a blowing fan

☐ Abs ☐ VMild ☐ Mild ☐ Mod. ☐ Sev. ☐ N/A ☐ RFR

15. **BODY SENSITIVITY TO OBJECT CONTACT -** Inhibited by physical contact with objects other than harness handle on back. Manifested by change in energy, mouthing the harness or other object, inhibited reaction or slowing down and/or avoiding or freezing when placing item over head or wearing items such as dog jackets or body of the harness, hypervigilance with foot placement, avoidance of tight spaces, focus 2-3 feet in front of the dog

☐ Collar pressure ☐ Stepping on leaves, twigs, etc. ☐ Tight Spaces ☐ Head into harness ☐ Puddles

☐ Abs ☐ VMild ☐ Mild ☐ Mod. ☐ Sev. ☐ N/A ☐ RFR

Comments:

16. **ANXIOUS ABOUT RIDING IN VEHICLES -** Avoids getting into vehicle, does not settle when riding

☐ Abs ☐ VMild ☐ Mild ☐ Mod. ☐ Sev. ☐ N/A ☐ RFR

17. **INHIBITED OR PASSIVELY AVOIDANT WHEN EXPOSED TO POTENTIALLY STRESSFUL SITUATIONS -** Copes with stress poorly, evidenced passively such as shutting down, avoidance, withdrawal, displacement activity such as avoidant sniffing and/or quitting as a response to stress

☐ Abs ☐ VMild ☐ Mild ☐ Mod. ☐ Sev. ☐ N/A ☐ RFR

Comments:

18. **ACTIVATED WHEN EXPOSED TO POTENTIALLY STRESSFUL SITUATIONS -** Copes with stress poorly, evidenced by becoming more active such as faster movements, taking treats harder, more distracted or other displacement behaviors to release emotional energy

☐ Abs ☐ VMild ☐ Mild ☐ Mod. ☐ Sev. ☐ N/A ☐ RFR

Comments:

19. **EXCITABLE -** Easily activated in response to stimuli such as, but not limited to, greeting a person, seeing another animal, being petted, anticipating going outside or for a walk or car ride; may whine in response to stimulus

☐ Abs ☐ VMild ☐ Mild ☐ Mod. ☐ Sev. ☐ N/A ☐ RFR

Comments:

#	Item	Abs	VMild	Mild	Mod.	Sev.	N/A	RFR
20.	**SLOW TO RETURN TO PRODUCTIVE EMOTIONAL STATE** - It takes the dog a long time to recover its productive emotional state following exposure to an arousing or stressful stimulus situation.	☐	☐	☐	☐	☐	☐	☐
	Comments:							
21.	**FIDGETY WHEN HANDLER IS IDLE** - Unsettled and/or pursues own interests when handler is idle ☐ demand barking at handler	☐	☐	☐	☐	☐	☐	☐
	Comments:							
22.	**FEAR WHEN ON ELEVATED AREAS, DROP-OFFs, etc** - Dog is fearful, apprehensive, hesitant near platform edges, or other elevated areas	☐	☐	☐	☐	☐	☐	☐
	Comments:							
23.	**BARKS PERSISTENTLY** - Barks persistently when alarmed or excited	☐	☐	☐	☐	☐	☐	☐
	Comments:							
24.	**HIGH ENERGY LEVEL** - Requires more energy outlets than the average dog to achieve a calm demeanor	☐	☐	☐	☐	☐	☐	☐
	Comments:							
25.	**LACKS FOCUS** - Looking around; attention moves from one stimulus to another without maintaining focus on task; has trouble staying on task	☐	☐	☐	☐	☐	☐	☐
	Comments:							
26.	**MOVEMENT EXCITES** - Easily distracted by non-animal movement: blowing leaves, flashlight, hose spraying water, etc., and has difficulty redirecting attention	☐	☐	☐	☐	☐	☐	☐
	Comments:							
27.	**CHASING ANIMALS** - Excited by animals other than dogs (e.g. birds, insects, squirrels, etc.) with persistent interest	☐	☐	☐	☐	☐	☐	☐
	Comments:							
28.	**DOG DISTRACTION** - Persistent interest in and high excitability level with other dog(s)	☐	☐	☐	☐	☐	☐	☐
	Comments:							
29.	**SNIFFING** - Distracted by olfactory stimuli	☐	☐	☐	☐	☐	☐	☐
	Comments:							
30.	**SCAVENGES** - Scavenges for food or other items anytime	☐	☐	☐	☐	☐	☐	☐
	Comments:							
31.	**INAPPROPRIATE BEHAVIOR AROUND THE HOME** - Chews household items, steals food or other items, tries to take things off counter tops, tables, etc., or out of garbage can. Carries or moves household objects.	☐	☐	☐	☐	☐	☐	☐
	Comments:							
32.	**LACKS INITIATIVE-** Lacks intrinsic motivation to seek solutions while performing a task requested by its handler	☐	☐	☐	☐	☐	☐	☐
33.	**NOT WILLING** - Dog pursues own interests; lacks apparent desire to respond to handler	☐	☐	☐	☐	☐	☐	☐
	Comments:							
34.	**RESOURCE GUARDING TOWARD PEOPLE** - Exhibits signs of aggressive guarding or possessiveness of resources (objects, toys, food) toward people who are present and/or approaching	☐	☐	☐	☐	☐	☐	☐
35.	**AGGRESSION TOWARD STRANGERS** - Suspicious of unfamiliar people; raised hackles, growling, barking, baring of teeth when stranger approaches	☐	☐	☐	☐	☐	☐	☐
36.	**AGGRESSION TOWARD DOGS** - Shows aggression toward other dogs (charges, growls, rushes, bites or attempts to bite) **ON LEASH**	☐	☐	☐	☐	☐	☐	☐
	**OFF LEASH**	☐	☐	☐	☐	☐	☐	☐
	Comments:							
37.	**RESOURCE GUARDING TOWARD DOGS OR OTHER PETS** - Exhibits signs of aggressive guarding or possessiveness of resources (objects, toys, food) toward dogs or other pets that are present and/or approaching ☐ At home ☐ In Kennel	☐	☐	☐	☐	☐	☐	☐
38.	**INAPPROPRIATE ELIMINATION WHILE WORKING EN ROUTE**	☐	☐	☐	☐	☐	☐	☐
	Comments:							
39.	**SOCIALLY INAPPROPRIATE BEHAVIOR WITH PEOPLE-** Exhibits poor social manners with people	☐	☐	☐	☐	☐	☐	☐
	Comments:							
40.	**INCONSISTENT** - Dog's demeanor, confidence and ability to cope is inconsistent over multiple assessments in various environments	☐	☐	☐	☐	☐	☐	☐
	Comments:							

41. HANDLER/DOG TEAM: How well dog and handler work together

Very Connected……………….....Not Connected

☐ ☐ ☐ ☐ ☐ N/A ☐

Comments:

42. RELATIONSHIP SKILLS - Handler's ability to help team work well together and/or advance the dog's confidence and/or skills

☐ Poor Use of You ☐ Leash Manages ☐ Pressures Dog ☐ Confusing Communication

Very Skilled…………..……….....…Less Skilled

☐9 ☐8 ☐7 ☐6 ☐5 ☐4 ☐3 ☐2 ☐1

43.	**COMPARISON 9-1 SCORE -** How well suited is this dog for the work your organization does compared with our current dogs	☐9 ☐8 ☐7 ☐6 ☐5 ☐4 ☐3 ☐2 ☐1
	**General Comments:**	
44.	**SOCIALLY INAPPROPRIATE BEHAVIOR WITH DOGS -** dIsplays socially inappropriate behavior around dogs.	Abs ☐ VMild ☐ Mild ☐ Mod. ☐ Sev. ☐ N/A ☐ RFR ☐
	Comments:	
45.	**THUNDER REACTION-** Awareness and anxiety of thunderstorm	Abs ☐ VMild ☐ Mild ☐ Mod. ☐ Sev. ☐ N/A ☐ RFR ☐
	Comments:	
46.	**KENNELS POORLY -** Exhibits signs of inability to adapt to kenneling.	Abs ☐ VMild ☐ Mild ☐ Mod. ☐ Sev. ☐ N/A ☐ RFR ☐
	Comments:	
47.	**WORKING SPEED-** Pace at which the dog is walking while doing work the dog is trained to do.	Fast ☐ Mod- ☐ Mod ☐ Mod+ ☐ Fast ☐ N/A ☐
	Comments:	
48.	**GAIT WHEN MOVING OUT -** How easily the dog maintains a trot when moving quickly	1 -Trots Easilty ☐ 2-Can't Maintain ☐ 3-Difficulty Trotting ☐
	Comments:	
49.	**HOUSEBREAKING PROBLEMS-** Frequency at which dog eliminates in home	Abs ☐ VMild ☐ Mild ☐ Mod. ☐ Sev. ☐ N/A ☐ RFR ☐
	Comments:	
50.	**INNATE DESIRE TO WORK -** Lacks intrinsic drive and motivation to learn tasks and be engaged with the handler	Abs ☐ VMild ☐ Mild ☐ Mod. ☐ Sev. ☐ N/A ☐ RFR ☐
	Comments:	
51.	**AVOIDANCE OF EXHAUST FROM VEHICLES -** Exhibits avoidance when approaching exhaust from vehicles	Abs ☐ VMild ☐ Mild ☐ Mod. ☐ Sev. ☐ N/A ☐ RFR ☐
	Comments:	
52.	**ALERTS TO NOISE HEARING DOGS -** Exhibits unwanted behavior to noises	Abs ☐ VMild ☐ Mild ☐ Mod. ☐ Sev. ☐ N/A ☐ RFR ☐
	Comments:	

<u>Operational definitions for Behavior Checklist</u> – V6

1/27/18 Added definitions for items 47-52
1/27/2018 BCL item 15 Body sensitivity edited
11/7/18 2 Noise-clarification, 19-excitment-significant modification 47 work speed, more details
7/27/20 11 Body Handling clarification, 13 Harness Sensitivity – title clarification, 15 Body Sensitivity clarification, 21 Fidgety – significant change removed times, added handler intervention, 25 Focus – clarification, 31 Inappropriate behavior around the home – significant changes.
3/20/21 43 Comparison score-clarification
5/14/21 38 Inapprop Elimination clarification, Added Whines Excessively

1. **ANXIOUS IN UNFAMILIAR LOCATIONS -** Initially anxious during first visits to unfamiliar locations. Exhibited as increased or inhibited activity, cautious and/or less responsive or focused.

Absent – Unfazed by change in environments. Remains relaxed.

Very mild – Initial slight signs of anxiety in unfamiliar locations. Recovers very quickly and remains able to work and respond effectively. Signs may include mild tongue flicking, mild restlessness or slowing, slight loss of responsiveness to work.

Mild – Mild or moderate signs of anxiety in unfamiliar locations. Recovers quickly. Signs may include frequent tongue flicking, moderate restlessness or slowing, mild loss of responsiveness to work.

Moderate – Pronounced anxiety to unfamiliar locations and slow recovery. Signs may include moderate restlessness, panting, whining, fast jerky movements, inhibited, compressed, escape behavior, seeking some handler support. Maintains work with encouragement.

Severe – Very strong anxiety in unfamiliar locations. Fails to recover to previous level of composure and responsiveness. Signs may include tongue flicking, whining, fast jerky movements, escape behavior, inhibited, compressed, clinging to handler. Does not maintain work.

2. **NOISE SENSITIVITY -** Startle, tense body language, hurries, escape or displacement behaviors when exposed to noise such as car horns, hair dryers, vacuums, banging

Absent – No startle response, fear or apprehension when exposed to sudden or loud noises from any source other than thunder and traffic.

Very mild – Very mild signs of concern or startle when exposed to sudden or loud noises from any source other than thunder and traffic. Signs may include a very slight change in energy (slowing down or speeding up), briefly moves away with quick return and/or slightly tense body language with almost immediate return to normal. No concern noted if exposure repeated. Maintains work.

Mild – Mild signs of concern or startle when exposed to sudden or loud noises from any source other than thunder and traffic. Signs may include a mild to moderate change in energy (slowing down or speeding up), mild coping strategies of moving away or displacement sniffing and/or mild to moderate tense body language. Returns to normal readily with support or time to resolve. Maintains work and improves with subsequent exposure.

Moderate – Moderate concern or startle when exposed to sudden or loud noises from any source other than thunder and traffic that interferes with dog's ability to maintain work without encouragement. Signs may include a moderate change in energy (slowing down or speeding up) and/or moderate tense body language and slow to habituate. Dog may exhibit coping strategy of moving away, release of emotional energy and/or up to 1 minute of habituation time.

Severe – Severe concern or fear reaction when exposed to sudden or loud noises from any source other than thunder and traffic that interferes with dog's ability to maintain work despite encouragement. Signs may include a moderate to severe change in energy (slowing down or speeding up) and/or very tense body language. Dog may exhibit strong coping strategy of moving away, release of emotional energy and/or over 1 minute of habituation time despite support.

3. **FEAR OF NOVEL OBJECTS** - Fearful, avoidant, or suspicious of unfamiliar objects, which could be anything, but common examples are plastic bags, statues, yard equipment, balloons, etc.

Absent – No startle response, fear or apprehension when exposed to unfamiliar objects. Will readily approach and investigate novel objects if allowed to.

Very mild – Very mild signs of concern or startle when exposed to unfamiliar objects. Signs may include a very slight change in energy (slowing down or speeding up) and/or slightly tense body language with almost immediate return to normal. No concern noted if exposure repeated and will generally approach and investigate object once over initial concerns. Maintains work.

Mild – Mild signs of concern or startle when exposed to unfamiliar objects. Signs may include a mild to moderate change in energy (slowing down or speeding up) and/or mild to moderate tense body language with rapid return to normal. Shows mild reluctance to approach and investigate novel objects but will do so with encouragement. Maintains work and improves with subsequent exposure.

Moderate – Moderate concern or startle when exposed to unfamiliar object that interferes with dog's ability to maintain work without encouragement. Signs may include a moderate change in energy (slowing down or speeding up) and/or moderate tense body language and slow to habituate. Shows some reluctance to approach novel objects even with encouragement. Dog may exhibit coping strategy of moving away slightly or releasing emotional energy by increased activity and/or up to 1 minute of habituation time.

Severe – Severe concern or fear reaction when exposed to unfamiliar object that interferes with dog's ability to maintain work despite encouragement. Signs may include a moderate to severe change in energy (slowing down or speeding up) and/or very tense body language. Shows strong reluctance to approach novel objects despite encouragement. Dog may exhibit strong coping strategy of moving away or releasing emotional energy by increased activity and/or over 1 minute of habituation time despite support.

4. **FEAR OF UNDERFOOTINGS** – Fearful, nervous, apprehensive of various walking surfaces: slippery floors, gratings, other

Absent – No fear or apprehension when approaching different walking surfaces (slippery floors, gratings); crosses over them without changing pace.

Very mild – Very mild signs of avoidance when approaching or walking on different surfaces (slippery floors, gratings). Signs may include slight hesitancy to step on, very mild hurrying across surface, and mildly tense body language. Maintains work.

Mild – Mild signs of avoidance when approaching or walking on different surfaces. Signs may include mild hesitancy to step on with mild transient compression, mild hurrying across surface, and moderately tense body language. Maintains work and improves with subsequent exposure without encouragement from handler.

Moderate – Moderate signs of avoidance when approaching or walking on different surfaces. Signs may include balking on approach, attempts to jump over, hurrying to get off surface or slowing down dramatically. Will always tend to avoid them, but not at the expense of making a clearance error or endangering the person. Requires encouragement to maintain work.

Severe – Fearful when approaching or walking on different surfaces. Signs may include hesitancy, hurrying, balking or refusal, very tense body language. Does not maintain work. Will avoid them even if it results in a clearance error or other mistake that may endanger the person.

5. **FEAR OF DOGS** – Inhibited response to other dogs on leash or off leash such as low body posture, pulling back, head low, apprehensive, avoidant, hackles, withdraws, reluctant to proceed, watches

Absent – Shows no signs of fear or suspicion of unfamiliar dogs. Comfortable around other dogs.

Very mild – Slightly cautious when approached by isolated unfamiliar dogs but quickly refocuses on work with verbal encouragement. Signs may include very mildly raised hackles, very mild slowing down, tail low, and/or slight avoidance. Remains responsive to handler and recovers immediately

Mild – Occasionally mild cautious around other dogs. Signs may include mildly raised hackles, mild slowing down, moving away from and/or avoiding an approaching dog and may briefly vocalize. Responds readily to handler intervention and recovers quickly once past the dog.

Moderate – Moderately cautious around other dogs. Signs may include moderately raised hackles, low body posture, pulling back, head low, watching other dog closely but typically without facing them directly and/or repeated vocalizations. Requires a lot of handler support in the presence of other dogs.

Severe – Very cautious or fearful of other dogs and typically avoids them. When exposed to dogs, signs may include stress and/or fear (very raised hackles, tongue flicking, trembling, growling, retreat, escape). May become aggressive when in a situation where it cannot easily escape ('cornered'). Requires a lot of handler support in the presence of other dogs.

6. **FEAR OF STAIRS** - Hurries, refuses, or hesitant on stairs: closed stairs, open back stairs, open grate stairs, upstairs, downstairs

Absent – Is relaxed getting on or going up and down stairs of all types.

Very mild – Very mild signs of avoidance when approaching or walking on stairs. Signs may include slight hesitancy to step on, very mild hurrying on stairs, and very mild tense body language. Maintains work.

Mild – Mild signs of avoidance when approaching or walking on stairs. Signs may include mild hesitancy to step on with mild transient compression, mild hurrying on stairs, and mildly tense body language. Maintains work and improves with subsequent exposure.

Moderate – Moderate signs of avoidance or concern when approaching or walking on stairs. Signs may include balking on approach, attempts to jump off, hurrying up then whining and hesitant to go down, placing front feet on stairs but not rear feet at first then getting on stairs, compresses when moving on stairs, rushes to exit. Is not comfortable matching speed to handler or waiting on stairs.

Severe – Fearful when approaching or walking on stairs. Signs may include hesitancy, hurrying, balking or refusal, very tense body language. Does not maintain work. Will avoid stairs or hurry when moving on them even if it results in a clearance error or other mistake that may endanger the person. Rushes to exit.

7. FEAR OF TRAFFIC - Tense body language, tail low or tucked, change in activity level, startle, retreat, escape in response to sight and sounds of vehicle traffic

Absent – Is comfortable and at ease around traffic regardless of intensity.

Very mild – Slightly apprehensive around heavy or noisy traffic. Recovers quickly. Signs may include mild tongue flicking, and/or mildly tense body language (ears low & pulled back slightly, lips pulled back). Maintains work.

Mild – Mildly apprehensive or distressed around heavy or noisy traffic. Signs may include very mild hurrying to walk away from area with good recovery, frequent tongue flicking, transient mild panting, and/or moderately tense body language. Maintains work and improves with subsequent exposure.

Moderate – Moderate signs of apprehension or distress around heavy or noisy traffic. Signs may include moderate hurrying to walk away from area, tense body language, startle with quick recovery but not retreat. Loses focus on work but handler can support dog through it.

Severe – Fearful around traffic. Signs may include tense body language, retreat, startle with slow recovery. Cannot perform its work and tries to escape.

8. **SEPARATION ANXIETY** - Restless, vocalizes, and/or becomes destructive when left alone

Absent – Shows no signs of distress when left alone.

Very mild – Very mild whining and/or is very mildly restless initially when left alone but settles and quiets within 5 minutes.

Mild - Mild whining and/or a few barks and/or is mildly restless when left alone but settles and quiets eventually within 5 minutes.

Moderate – Moderate whining and/or barking and/or moderately agitated and restless when left alone. Vocalizes throughout the duration of separation.

Severe – Very vocal and/or very agitated and distressed when left alone. May be destructive or self-mutilates.

9. **HYPER-ATTACHMENT** - Overly attached to primary handler, seeks to return to primary handler when handled by others

Absent – Readily adapts to new handler or caretaker even when primary handler is visible.

Very mild – Demonstrates slight attachment to primary handler and familiar individuals; noted by looking for handler, but readily adapts to a new handler or caretaker who provides support for short periods.

Mild – Demonstrates mild attachment to primary handler and familiar individuals; noted by looking for handler or exhibiting mild anxiety with or without minor vocalizing, but readily adapts to new handler or caretaker with support, which may include use of food.

Moderate – Forms strong attachments to primary handler and familiar individuals. Adapts slowly to new handler or caretaker even when primary handler is not visible; noted by prolonged seeking of primary handler and/or moderate anxiety and difficulty and/or unwillingness to work with and respond to new handler despite providing support. Difficulty staying on task.

Severe – Forms very strong attachments to primary handler and familiar individuals. Very slow adaptation to new handler or caretaker if primary handler is not present; noted by severe anxiety and prolonged inability or unwillingness to work with and respond to new handler despite providing support. Unable to stay on task.

10. **FEAR OF STRANGERS** - Fearful, nervous, apprehensive with strangers, cautious, avoidant, hackling, slow approach, keying in, barking, growling, escape

Absent – Shows no signs of fear or suspicion of unfamiliar people. Comfortable around strangers regardless of strange appearance or behavior.

Very mild – Slightly cautious when approached by or moving past isolated unfamiliar people. When approached, signs may include turning head away looking in other direction and/or leaning body away slightly. When moving past, signs may include watching person and/or slightly slowing down. Quickly recovers however may need a little verbal encouragement from handler

Mild – Mildly cautious when approached by or moving past isolated unfamiliar people. When approached, signs may include moving body away to avoid contact and/or slight compression of body. When moving past, signs may include mild slowing down, mild focus on person, mild hackling, and/or mild compression of the body. Quickly recovers once past the person however may need support and encouragement from the handler.

Moderate – Moderately cautious when approached by or moving past isolated unfamiliar people. When approached, signs may include low body posture, moderately pulling back to avoid contact, moderate raised hackles and/or may vocalize. When moving past, signs may include moderately slowing down and/or hesitancy to pass by, moderate focus on person, moderate raised hackles, vocalization, and/or moderate compression of the body. Slower to recover even with support from the handler.

Severe – Very cautious when approached by or moving past isolated unfamiliar people. When approached, signs may include dilated eyes, very raised hackles, trembling, vocalizing, retreat and/or escape. May become aggressive in a situation where it cannot easily escape ('cornered'). Is unable to work past person, may vocalize and/or severe compression of the body. Very slow to recover even with support from the handler.

11. **BODY HANDLING CONCERN** – Avoidant, anxious, fearful, and/or aggressive when handled for non-invasive activities in a manner typical of a vet exam, grooming and/or nail clipping

Absent – Relaxed when handled for non-invasive activities in a manner typical of a vet exam, grooming, nail clipping

Very mild – Very mild signs of concern such as very mild tongue flicking, very mildly tense body language and muscle tone and/or yawning when handled for non-invasive activities in a manner typical of a vet exam, grooming, nail clipping. Is not difficult to manage and needs no or minimal supportive handling.

Mild – Mild concern displayed by becoming mildly activated or inhibited and may have mild tongue flicking, mildly tense body language and muscle tone, when handled for non-invasive activities in a manner typical of a vet exam, grooming, nail clipping. Able to tolerate exam with mild supportive handling.

Moderate – Moderate concern displayed by becoming moderately activated or inhibited and typically strong struggling, pulling back or flailing when handled for non-invasive activities in a manner typical of a vet exam, grooming, nail clipping. Very difficult to manage but can with significant effort finish exam.

Severe – Very fearful of body handling displayed by becoming severely activated or inhibited. Behaviors may include escaping or attempting to escape even when on floor, growling, having raised hackles, baring teeth, snapping and/or attempting to bite when handled for non-invasive activities in a manner typical of a vet exam, grooming, nail clipping. Very difficult to manage despite significant attempts to support and unable to effectively complete exam.

12. **RETREATS WHEN REACHED FOR** – Moves head or face away when reached for by familiar persons

Absent – Does not move head or face away when reached for by familiar persons.

Very mild – Very mildly moves head or face away when reached for by familiar persons. With a little encouragement, readily allows being reached for without a food lure.

Mild – Mildly moves head or face away when reached for by familiar persons. Readily responds to a food lure and/or encouragement not to retreat when reached for.

Moderate – Moderately moves head or face away when reached for by familiar persons. Hesitantly responds to a food lure and/or encouragement not to retreat when reached for.

Severe – Severely moves head or face away when reached for by familiar persons. Slowly responds to a food lure and/or encouragement but remains tentative or continues to retreat when reached for.

13. **HARNESS HANDLE ON BACK SENSITIVITY** - Drops rear quarters when harness handle lays on back

Absent – Dog does not demonstrate any drop in body when harness handle lies on its back vs. when handle is not on its back.

Very mild – Dog initially shows very mild crouching, dropping **no more than one quarter** lower than its normal walking height when the harness handle lies on its back and when working acclimates within a few minutes, resuming walking almost naturally.

Mild – Dog initially shows mild crouching, **dropping to no more than half** its natural walking height when harness handle lies on its back and when working acclimates within a few minutes, resuming walking almost naturally.

Moderate – Dog initially shows moderate crouching, **dropping very low to the ground** and walking in a deeply crouched position, but acclimates within a few minutes when working and resumes walking almost naturally.

Severe – Dog shows severe crouching, **dropping very low to the ground** and walking in a deeply crouched position with knees within a few inches of the ground when harness handle lies on back. **Acclimation to harness handle on back is very slow**.

14. **AVOIDANCE OF BLOWING FAN -** Fearful or avoidant when walking past a blowing fan

Absent – Unfazed walking by blowing fan. Remains relaxed.

Very mild – Initial very mild signs of anxiety walking closely past blowing fan. Recovers very quickly and remains able to work & respond effectively. Signs may include mild tongue flicking, mild restlessness, and/or slight loss of responsiveness to work.

Mild – Mild signs of anxiety walking closely past blowing fan. Recovers quickly. Signs may include frequent tongue flicking, moderate restlessness, slight hurrying, and/or mild loss of responsiveness to work.

Moderate – Moderate anxiety and slow recovery walking closely past blowing fan. Signs may include restlessness, panting, whining, fast jerky movements, moderately inhibited, compressed and/or seeking some handler support. Maintains work with encouragement.

Severe – Severe anxiety walking closely past blowing fan. Signs may include rushing, tongue flicking, whining, fast jerky movements, strongly inhibited, compressed. and/or clinging to handler. Does not maintain work. Fails to recover to previous level of composure and responsiveness.

15. **BODY SENSITIVITY TO OBJECT CONTACT** – Inhibited by physical contact with objects other than harness handle on back. Manifested by change in energy, mouthing the harness or other object touching the dog, inhibited reaction or slowing down and/or avoiding or freezing when placing item over head or wearing items such as dog jackets or body of the harness, hypervigilance with foot placement, avoidance of tight spaces, focus 2-3 feet in front of the dog.

Absent – Unfazed by physical contact with objects such as stepping on rough surfaces, leaves, twigs etc.; collar or leash touching body, placing head into harness, wearing harness or other objects, etc. Remains relaxed. If newly exposed, score after walking 1 minute to acclimate if needed

Very mild – Initial very mild signs of anxiety from physical contact with objects such as stepping on rough surfaces, leaves, twigs etc.; collar or leash touching body, placing head into harness, and/or wearing harness or other objects. Recovers very quickly and remains able to work & respond effectively without handler support. Anxiety signs may include infrequent tongue flicking, mild slowing down and/or very mild avoidance with a slight loss of responsiveness to work. If newly exposed score after walking 1 minute to acclimate if needed

Mild – Mild signs of anxiety from physical contact with objects such as stepping on rough surfaces, leaves, twigs etc.; collar or leash touching body, placing head into harness, and/or wearing harness or other objects. Recovers quickly with support or additional acclimation time but has mild loss of responsiveness to work. Anxiety signs may include frequent tongue flicking, mild hypervigilance where stepping, moderate slowing down or restlessness and/or mild avoidance of tight spaces. If newly exposed, score after walking 1 minute to acclimate if needed

Moderate – Moderate anxiety and slow recovery from physical contact with objects. Maintains work with encouragement but has moderate loss of responsiveness to work. Anxiety signs may include moderate hypervigilance when walking on rough surfaces, avoidance of tight spaces and/or moderately avoiding harness being placed over head and/or wearing harness or other objects. If newly exposed, score after walking 1 minute to acclimate if needed

Severe – Severe anxiety from physical contact with objects. Does not maintain work and fails to recover to previous level of composure and responsiveness despite significant handler support or acclimation time. Signs may include severe inhibition including freezing, hypervigilance when stepping, overriding the handler's instructions to avoid situation, and/or freezing or strongly avoiding harness being placed over head and/or wearing harness or other objects. If newly exposed, score after walking 1 minute to acclimate if needed

16. **ANXIOUS ABOUT RIDING IN VEHICLES** – Avoids getting into vehicle, does not settle when riding

Absent – Unfazed getting into and riding in cars and vans. Remains relaxed.

Very mild – Initial slight signs of stress related anxiety when getting into and riding in cars and vans. Recovers very quickly and remains able to work & respond effectively. Signs may include infrequent tongue flicking, mild restlessness, and/or slight loss of responsiveness to work.

Mild – Mild signs of stress related anxiety getting into and riding in cars and vans. Recovers quickly. Signs may include frequent tongue flicking, moderate restlessness, slight rushing, and/or mild loss of responsiveness to work.

Moderate – Moderate stress related anxiety and slow recovery getting into and riding in cars and vans. Signs may include very frequent tongue flicking, restlessness, panting, whining, fast jerky movements, inhibited, compressed, and/or seeking some handler support. Maintains work with encouragement.

Severe – Severe stress related anxiety getting into and riding in cars and vans. Fails to recover to previous level of composure and responsiveness. Signs may include rushing, very frequent tongue flicking, whining, fast jerky movements, inhibited, compressed, and/or clinging to handler. Does not maintain work.

17. **INHIBITED OR PASSIVELY AVOIDANT WHEN EXPOSED TO POTENTIALLY STRESSFUL SITUATIONS** – Copes with stress poorly, evidenced passively such as shutting down, avoidance, withdrawal, displacement activity such as avoidant sniffing and/or quitting as a response to stress

Absent – No evidence of inhibited behavior when exposed to potentially stressful situations. Remains relaxed.

Very mild – In a few isolated instances, becomes very mildly inhibited in response to stress. The signs may include very mildly slowing down, short periods of avoidance sniffing and/or very mild tongue flicking. Responds readily to encouragement and recovers quickly. Does not interfere with its ability to work or continue responding to handler.

Mild – Occasionally becomes mildly inhibited in response to stress. The dog mildly internalizes stress. Signs may include mildly slowing down and mild withdrawing, mild panting, longer periods of avoidance sniffing and/or other avoidant responses and/or mild tongue flicking. Recovers reasonably quickly with encouragement or allowing time to process. Does not interfere with its ability to work or continue responding to handler.

Moderate – Becomes moderately inhibited in response to stress sometimes to the point of interfering with ability to work or continue responding to handler despite support and encouragement and/or allowing a short time to process. The dog moderately internalizes stress. Signs may include moderately slowing down and withdrawing, moderate panting, prolonged periods of avoidance sniffing and/or other avoidant responses.

Severe – Becomes severely inhibited in response to stress to a point of being unable to work or continue responding to handler despite support and encouragement and/or allowing a short time to process. The dog severely internalizes stress. Signs may include shutting down, severely withdrawing, prolonged and/or severe panting, prolonged periods of moderate tongue flicking, avoidance sniffing and/or other avoidant responses with inability to respond to handler.

18. **ACTIVATED WHEN EXPOSED TO POTENTIALLY STRESSFUL SITUATIONS** – Copes with stress poorly, evidenced by becoming more active such as faster movements, taking treats harder, more distracted, or other displacement behaviors to release emotional energy.

Absent – No evidence of animated behavior when exposed to potentially stressful situations.

Very mild – In a few isolated instances, becomes very mildly animated in response to stress. Signs may include very mild increase in body movements, very mild tongue flicking very mild display of displacement activities such as but not limited to short periods of scavenging, sniffing. Recovers very quickly with encouragement and/or allowing a short time to process. Does not interfere with its ability to work or continue responding to handler.

Mild – Occasionally becomes mildly animated in response to stress. Signs may include mild increase in body movements, mild tongue flicking, rare shaking of the head and/or scratching the body, mild panting, and/or mild display of displacement activities such as but not limited to repeated scavenging or attempts to scavenge. Recovers reasonably quickly with encouragement and/or allowing a short time to process. Does not interfere with its ability to work or continue responding to handler.

Moderate – Becomes moderately animated in response to stress sometimes to the point of interfering with ability to work or continue responding to handler despite support and

encouragement and/or allowing a short time to process. Signs may include moderate increase in body movements, some jerky body movements, running into things or showing less control of the body, moderate tongue flicking, occasional shaking of the head and/or scratching the body, moderate panting, and/or moderate display of displacement activities such as but not limited to repeated scavenging or attempts to scavenge.

Severe – Becomes hyper-animated in response to stress to a point of being unable to work or continue responding to handler despite support and encouragement and/or allowing a short time to process. Signs may include severe increase in body movements, very jerky body movements, running into things or showing more frequent poor control of the body, very frequent tongue flicking, frequent shaking of the head and/or scratching the body, severe or prolonged panting and/or severe display of displacement activities such as but not limited to repeated scavenging or attempts to scavenge.

19. **EXCITABLE** – Increases energy and arousal levels without observed stress signals in response to stimuli such as, but not limited to, greeting a person, seeing another animal, being petted, anticipating going outside or for a walk or car ride; may whine in response to stimulus

Absent – Remains calm in presence of exciting stimuli. No additional handler direction required to manage dog.

Very mild- Very mildly or mildly increases energy and arousal levels without observed stress signals in response to stimuli. Excitability does not interfere with working or responding to the handler when provided minimal encouragement, supportive direction and/or up to 15 seconds to process without handler intervention. Signs may include very brief very mild to mildly increased body movements such as but not limited to brief attempts of non-forceful jumping, pawing, or muzzling.

Mild – Moderately increases energy and arousal levels without observed stress signals in response to stimuli. Excitability does not interfere with working or responding to the handler when supportive direction is provided or allowing up to 30 seconds to process without handler intervention. Signs may include brief bursts of moderately increased body movements such as but not limited to light panting, 1 or 2 brief barks, repeated but controllable attempts of jumping, pawing and play biting,

Moderate – Moderately increases energy and arousal levels without observed stress signals in response to stimuli. Excitability interferes with working or responding to the handler despite supportive direction. The dog is difficult to manage and/or requires over 30 seconds but less than 2 minutes to return to a productive state even with supportive handling. Signs may include moderate increase in body movements, short periods of jerky body movements or one instance of running into things or showing less control of the body, moderate panting, prolonged or repeated barking that can be redirected.

Severe – Becomes wildly excited in response to stimuli to a point of being unable to work or continue responding to handler despite moderate to high levels of supportive direction. The dog is difficult to manage and/or requires over 2 minutes to return to a productive state even with supportive handling. Signs may include severe increases in body movements, repeated periods of jerky body movements, running into things or showing less control of the body, moderate to severe panting, prolonged or repeated behaviors such as barking, jumping, play biting that are very difficult or unable to be managed or redirected.

20. **SLOW TO RETURN TO PRODUCTIVE EMOTIONAL STATE** – It takes the dog a long time to recover its productive emotional state following exposure to an arousing or stressful stimulus situation.

Absent – Returns immediately (in less than 15 seconds) to productive emotional state after an exciting or stressful stimulus.

Very mild – Returns very quickly (15 to 30 seconds) to productive emotional state after an exciting or stressful stimulus has passed.

Mild – Returns quickly (30 seconds to 2 minute) to productive emotional state after an exciting or stressful stimulus has passed.

Moderate – Slow to return (2 to 5 minutes) to productive emotional state following exposure after an exciting or stressful stimulus has passed. May also exhibit progressive increase in recovery time following repeated exposure to exciting and/or stressful stimuli.

Severe – Very slow to return (> 5 minutes) to productive emotional state after an exciting or stressful stimulus has passed and often exhibits progressive increase in recovery time following repeated exposure to exciting and/or stressful stimuli.

21. **FIDGETY WHEN HANDLER IS IDLE** – Unsettled and/or pursues own interests when handler is idle

Absent – Waits patiently in a settled manner while handler is idle. Settles very quickly or immediately.

Very mild – Very mildly active and/or very mildly pursues other interests when handler is idle. Settles quickly without the need for handler intervention.

Mild – Mildly active and/or mildly pursues other interests, or briefly seeking handler interaction, requiring mild handler intervention to settle and wait patiently, when handler is idle.

Moderate – Moderately active and/or moderately pursues other interests or demanding interaction from handler requiring moderate handler intervention to settle and wait patiently, when handler is idle.

Severe – Hyperactive and/or strongly pursues other interests, or persistently and/or forcefully seeks handler interaction. Despite significant and repeated attempts at handler intervention, dog is unable to settle and wait patiently when handler is idle

22. **FEAR ON ELEVATED AREAS, DROP-OFFs, etc**- Dog is fearful, apprehensive, hesitant near platform edges or other elevated areas

Absent – Is relaxed when on elevated areas of all types.

Very mild – Very mild signs of caution when approaching edges or when on an elevated area. Signs may include very mild tense body language, hesitancy but maintains work.

Mild – Mild signs of apprehension when approaching edges, drop-offs, or when on an elevated area. Signs may include mildly tense body language, mild hesitancy with mild transient compression and/or mild hurrying. Maintains work and improves with subsequent exposure.

Moderate – Moderate signs of fear when approaching edges, drop-offs, or when on an elevated area. Signs may include moderately tense body language, balking on approach, pulling away from the edge, moderate compression. Is not comfortable matching speed to handler or waiting in the elevated area.

Severe – Severe signs of fear when approaching edges, drop-offs, or when on an elevated area. Signs may include severely tense body language, severe balking, or refusal on approach, severe pulling away from the edge, severe compression. Does not maintain work. Will not work effectively on elevated areas.

23. **BARKS PERSISTENTLY** - Barks persistently when alarmed or excited

Absent – Never or rarely barks when alarmed or excited.

Very mild – Occasionally will bark a few times when excited or alarmed but quiets immediately with no intervention needed from the handler and while still in presence of exciting/alarming stimulus.

Mild – Occasionally will bark a few times when excited or alarmed but requires handler direction and/or support to quiet quickly while still in presence of exciting/alarming stimulus.

Moderate – Often barks repeatedly when excited or alarmed and typically requires repeated direction and/or support from handler to stop barking while in the presence of an exciting/alarming stimulus.

Severe – Often barks repeatedly when excited or alarmed, despite repeated support from handler and/or efforts to redirect the behavior. Will continue to bark while the exciting/alarming stimulus is present.

24. **HIGH ENERGY LEVEL** - Requires more energy outlets than the average dog to achieve a calm demeanor

Absent – Requires minimal exercise or activity; may prefer to be sedentary. Content to lie down calmly for much of the day. Settles down quickly after a period of activity.

Very mild – Requires average exercise and activity to maintain a calm demeanor. May need only one short exercise period daily. Occasionally needs additional energy outlets to settle down and lie calmly with short periods of chewing toys or activity through the day.

Mild – Requires above average exercise to maintain a calm demeanor. Frequently needs additional energy outlets to settle down and lie calmly with short periods of chewing toys or activity through the day.

Moderate – Requires above average exercise and is frequently unable to maintain a calm demeanor despite exercise. Frequently needs atypically large amounts of energy outlets to settle down and does not consistently lie calmly with short periods of chewing toys or activity through the day.

Severe – Requires above average exercise and is rarely able to maintain a calm demeanor despite exercise. Consistently needs atypically large amounts of energy outlets to settle down and does not consistently lie calmly with short periods of chewing toys or activity through the day.

25. LACKS FOCUS – Looking around; attention moves from one stimulus to another without maintaining focus on an activity. This is not a measure of ‘engagement’, which is scored in number 41 Handler/Dog Team

Absent – Dog demonstrates a strong ability to concentrate in all types of environments or work situations.

Very mild – Dog demonstrates good ability to concentrate in all types of environments or work situations and is readily able t. May rarely shift attention for brief periods and is readily able to regain focus with minimal support from the handler.

Mild – Dog demonstrates good ability to concentrate in all types of environments or work situations. Occasionally shifts attention and requires mild directive actions or other support from handler to regain focus.

Moderate – Dog demonstrates poorer ability to concentrate in some environments or work situations. Frequently shifts attention and requires moderate directive action or other support from the handler to regain focus.

Severe – Dog demonstrates poor ability to concentrate in some environments or work situations. Very frequently shifts attention, moving from diversion to diversion and regularly requires directive action or other support from handler to regain focus or is unable to regain focus

26. **MOVEMENT EXCITES -** Easily distracted by non-animal movement: blowing leaves, flashlight, hose spraying water, etc., and has difficulty redirecting attention

Absent – Dog may ignore or briefly alert to movement such as light, leaves blowing, etc. and/or may briefly investigate or calmly follow movement when not working but does not interfere with work. Dog does not exhibit signs of excitement evidenced by increased activity, vocalizing.

Very mild – Dog alerts to and exhibits very mild interest in movement such as light, leaves blowing, etc. and may get very mildly excited such as very mild increased activity if not directed otherwise. Settles readily and remains responsive to handler.

Mild – Dog alerts to and shows easily interruptible, mild interest in movement such as light, leaves blowing, etc. and may get mildly excited such as mild increased activity, mild persistence and/or brief whining. May require 15 seconds or less to settle after stimulus is gone. Responds readily to direction from handler.

Moderate – Dog alerts to and exhibits moderate interest in movement such as light, leaves blowing, etc. resulting in moderate excitement such as moderately increased activity, decreased responsiveness to handler interfering with work, moderate whining and/or mild barking and/or moderate persistence. May require up to 3 minutes to fully settle after stimulus is gone.

Severe – Exhibits a high level of interest in movement such as light, leaves blowing, etc. and excitement and/or very persistent interest in movement which interferes with work. May require a prolonged time to fully settle after stimulus is gone.

27. **CHASING ANIMALS** - Excited by animals other than dogs (e.g. birds, insects, squirrels, etc.) with persistent interest

Absent – Largely ignores other animal (non-dog) distractions such as birds, squirrels, rabbits, etc. No “prey drive.” Remains focused on work regardless of movements in the environment.

Very mild – Exhibits very mild distraction and interest in chasing or lunging at other animals (non-dog) such as birds, squirrels, rabbits, etc. and quickly refocuses on work with encouragement without handler intervention and settles readily even in presence of animals.

Mild – Exhibits mild distraction and interest in chasing or lunging at other animals (non-dog) such as birds, squirrels, rabbits, etc. and remains responsive to handler. Able to refocus on work with mild handler intervention, does not exhibit persistent interest and settles readily even in presence of animals.

Moderate -- Exhibits moderate distraction and interest in chasing or lunging at other animals (non-dog) such as birds, squirrels, rabbits, etc. becoming less responsive to handler for brief periods. Exhibits mildly persistent interest but will readily settle with increased distance.

Severe – Exhibits severe distraction and persistent interest in chasing or lunging at other animals (non-dog) such as birds, squirrels, rabbits, etc. and is poorly responsive to handler. Difficult to regain focus on work and quickly returns attention to the chasing animals. Unable to maintain work focus.

28. **DOG DISTRACTION** - Persistent interest in and high excitability level with other dog(s)

Absent – Shows little or no interest in other dogs and focus is unaffected by their presence. Requires no intervention and/or support from handler.

Very mild – A few instances of losing focus and becoming very mildly distracted by another dog but quickly refocuses with very mild handler intervention and/or support.

Mild – Occasionally loses focus and becomes mildly distracted by another dog. Requires mild handler intervention and/or support to regain focus.

Moderate – Frequently loses focus and becomes moderately distracted around other dogs and/or exhibits persistent interest in other dogs. Usually requires moderate handler intervention and/or support to regain focus.

Severe – Very dog oriented. Consistently distracted around other dogs with persistent interest. Difficult or unable to regain focus despite handler intervention.

29. **SNIFFING -** Distracted by olfactory stimuli

Absent – Largely ignores olfactory stimuli or exhibits brief sniffing when not under command. Remains focused on work. Does not exhibit persistent interest.

Very mild – Briefly distracted by olfactory stimuli and exhibits very mild sniffing but quickly refocuses on work with encouragement. Able to refocus on work with very mild handler intervention. Does not exhibit persistent interest.

Mild – Mildly distracted by olfactory stimuli and exhibits mild sniffing but remains responsive to handler. Able to refocus on work with mild handler intervention. Does not exhibit persistent interest.

Moderate – Moderately distracted by olfactory stimuli and exhibits moderate sniffing becoming less responsive to handler for brief periods of 10 seconds or less and requires moderate handler intervention. Exhibits moderately persistent interest.

Severe – Highly distracted by olfactory stimuli and exhibits excessive sniffing becoming less responsive to handler for periods greater than 10 seconds requiring significant handler intervention. Difficult to regain focus on work and quickly returns attention to sniffing. Exhibits very strong persistent interest.

30. **SCAVENGES** - Scavenges for food or other items anytime

Absent – Never scavenges at any time or in any location.

Very mild – Scavenges in a few isolated instances but stops with a verbal cue and is easy to control. Is not persistent.

Mild – Occasionally scavenges but stops with a handler intervention and/or support. Is not persistent.

Moderate – Frequently scavenges and requires a high level of effort to redirect the behavior, but stops eventually with handler intervention and/or support. Is persistent.

Severe – Routinely scavenges and often returns to scavenging after handler intervention and/or support. Is persistent.

31. **INAPPROPRIATE BEHAVIOR AROUND THE HOME** – Chews household items, steals food or other items, tries to take things off counter tops, tables, etc. or out of garbage can. Carries or moves household objects.

Absent – Can be reliably trusted not to chew household items, take things from counters, take garbage or get on the furniture in familiar places, and easily adapts to new places.

Very mild – May take or move a household object but responds immediately to verbal guidance. Is not persistent and never destructive with these items. Does not take items from the garbage. May put feet on furniture but responds readily to handler guidance and does not persist. May invade human personal space or sniff counter or table due to interest in food but makes no attempt to lick or take it or put feet or head over the counter or table.

Mild – Attempts to get on furniture or move household items but is not destructive and will not counter surf. The dog might play a little "keep away" (taking an object and moving away/running off with it), but it is not persistent and will give up the item readily. The dog may check counters and garbage cans but will not jump up or knock over.

Moderate – Dog has done any of these things more than once in the past month: Been mildly destructive with household items and/or handler has mild difficulty getting the dog to drop the item or stop playing 'keep away'. Jumped on counter and requires repeated guidance from handler to stop attempting this. Attempted to grab food or items from counters or garbage, or has knocked the garbage over, and requires more than mild handler direction and/or is persistent.

Severe – Dog has done one or more of these things more than once in the past month: Been moderately or severely destructive of household items, and/or bene moderately persistent when playing "keep away" and will not give up the item readily. Jumped on furniture and/or is persistent in attempting to jump on furniture despite repeated guidance. Attempted to grab food or items from counters or garbage cans or has knocked garbage over.

32. **LACKS INITIATIVE -** Lacks intrinsic motivation to seek solutions while performing a task requested by its handler

Absent – Ideal intrinsic motivation to seek solutions while performing a task requested by its handler.

Very mild – Very mild lack of intrinsic motivation to seek solutions while performing a task requested by its handler.

Mild – Mild lack of intrinsic motivation to seek solutions while performing a task requested by its handler.

Moderate - Moderate lack of intrinsic motivation to seek solutions while performing a task requested by its handler.

Severe – Severe lack of intrinsic motivation to seek solutions while performing a task requested by its handler.

33. **NOT WILLING** - Dog pursues own interests; lacks apparent desire to respond to handler

Absent – Dog typically exhibits strong desire to comply with handler's direction.

Very Mild - Dog occasionally pursues own interests but does so very mildly. Typically exhibits moderate desire to comply with handler's direction.

Mild - Dog frequently pursues own interests but does so mildly. Typically exhibits mild desire to comply with handler's direction.

Mod - Dog frequently pursues own interests and does so moderately. Typically exhibits a low desire to comply with handler's direction.

Severe - Dog very frequently pursues own interests and does so moderately to severely. Typically exhibits a very low desire to comply with handler's direction

34. **RESOURCE GUARDING TOWARD PEOPLE - Exhibits signs of aggressive guarding or possessiveness of resources (objects, toys, food) toward people who are present and/or approaching**

Absent – Shows no signs aggressive guarding or possessiveness of resources (objects, toys, food) toward people who are approaching and/or attempting to take away an item.

Very mild – Hovers over objects/toys/food without vocalizing when people are approaching or attempting to take away an item. May also exhibit very slight signs of conflict with change in energy, tongue flicking, avoidance, eating more quickly or stop eating but yields almost immediately on own.

Mild – Hovers over objects/toys/food when people are approaching or attempting to take away an item. May also exhibit moderate signs of conflict moving away from person, tongue flicking and/or brief stiffening, brief staring with or without half-moon shape of the whites of the eyes showing (whale eye), very brief growling but yields readily with handler direction.

Moderate – Hovers over objects/toys/food and displays overt aggressive signs when people are approaching and/or attempting to take away an item. Aggressive signs may include prolonged stiffening and/or staring with half-moon shape of the whites of the eyes showing (whale eye), growling, any baring of teeth and does not always yield with efforts to redirect the behavior.

Severe – Hovers over objects/toys/food and displays overt aggressive signs when people are approaching and/or attempting to take away an item. Aggressive signs may include prolonged stiffening and/or staring with half-moon shape of the whites of the eyes showing (whale eye) AND growling, any baring of teeth and attempts to bite or snap when challenged.

35. **AGGRESSION TOWARD STRANGERS** - Suspicious of unfamiliar people; raised hackles, growling, barking, baring of teeth when stranger approaches

Absent – Shows no signs of aggression or distrust toward strangers.

Very mild – Very mild signs of distrust toward some strangers. Signs may include occasional suspicious focus on certain unfamiliar individuals and/or raised hackles. Quickly refocuses on work with verbal encouragement.

Mild – Mild signs of distrust toward some strangers. Signs may include occasional suspicious focus on certain unfamiliar individuals and or raised hackles, together with muted barking or growling. Readily refocuses on work with encouragement.

Moderate – Moderate signs of aggression toward some strangers. Signs may include frequent suspicious focus on unfamiliar individuals, together with raised hackles and growling, barking and/or baring teeth when person approaches too quickly, or appears threatening.

Severe – Highly aggressive toward most strangers, including frequent suspicious focus on unfamiliar individuals, together with raised hackles, and growling, barking and/or baring teeth when person approaches. May bite or attempt to bite if approached by a stranger and/or may try to chase the person away.

36. **AGGRESSION TOWARD DOGS** - Shows aggression toward other dogs (charges, growls, rushes, bites or attempts to bite)

Absent – Shows no signs of aggression toward unfamiliar dogs.

Very mild – Very mild signs of aggressive tendencies toward some unfamiliar dogs. Signs include very mildly raised hackles, very mildly stiff body language and/or very mild profiling in the presence of unfamiliar dogs. Does not growl, bark, bare teeth or attempt to bite. Readily refocuses on work with very mild handler support and/or encouragement.

Mild – Mild signs of aggressive tendencies toward some unfamiliar dogs. Signs include mildly raised hackles, mildly stiff body language and/or mild profiling in the presence of unfamiliar dogs, together with muted growling and/or barking in the presence of unfamiliar dogs. Does not bare teeth or attempt to bite. Readily refocuses on work with mild handler support and/or encouragement.

Moderate – Moderate signs of aggressive tendencies toward some or many unfamiliar dogs. Signs include moderately raised hackles, moderately stiff body language and/or moderate profiling in the presence of unfamiliar dogs, charges, rushes, often while growling, barking and/or baring teeth. Does not attempt to bite when in presence of unfamiliar dog. Moderately difficult to refocus on work with significant handler intervention and support.

Severe – Highly aggressive toward some or many unfamiliar dogs. Signs include severe raised hackles, severe stiff body language and/or severe profiling in the presence of unfamiliar dogs. Charges, rushes, often while growling, barking, baring teeth and/or bites or attempts to bite when in presence of unfamiliar dog. Is very difficult to refocus on work with significant handler intervention and support.

37. **RESOURCE GUARDING TOWARD DOGS OR OTHER PETS - Exhibits signs of aggressive guarding or possessiveness of resources (objects, toys, food) toward dogs or other pets that are present and/or approaching**

Absent – Shows no signs of aggressive guarding or possessiveness of resources (objects, toys, food) toward dogs or other pets that are approaching and/or attempting to take away an item.

Very mild – Hovers over objects/toys/food without vocalizing when dogs or other pets are approaching or attempting to take away an item. May also exhibit very slight signs of conflict with change in energy, tongue flicking, avoidance, eating more quickly or stop eating but yields almost immediately on own.

Mild – Hovers over objects/toys/food when dogs or other pets are approaching or attempting to take away an item. May also exhibit as moderate signs of conflict moving away, tongue flicking and/or brief stiffening, brief staring with or without half-moon shape of the whites of the eyes showing (whale eye), very brief growling but yields readily with handler direction.

Moderate – Hovers over objects/toys/food and displays overt aggressive signs when dogs or other pets are approaching and/or attempting to take away an item. Aggressive signs may include prolonged stiffening and/or staring with half-moon shape of the whites of the eyes showing (whale eye), growling, any baring of teeth and does not always yield with efforts to redirect the behavior.

Severe – Hovers over objects/toys/food and displays overt aggressive signs when dogs or other pets are approaching and/or attempting to take away an item. Aggressive signs may include prolonged stiffening and/or staring with half-moon shape of the whites of the eyes showing (whale eye) AND growling, any baring of teeth and attempts to bite or snap when challenged.

38. **INAPPROPRIATE ELIMINATION WHILE WORKING EN ROUTE**

Absent – Eliminates on route only at the curb or specified location, on command

Very mild – Rarely eliminates en route without handler command and the tendency to do so has improved substantially with age and is unlikely to present a problem in the future.

Mild – Occasionally eliminates en route without handler command. Adjustment of the feeding schedule/amount and diligent relieving schedule has improved the problem.

Moderate – Often eliminates or attempts to eliminate en route without handler command. Has shown some improvement with age and/or changes in feeding routine.

Severe – Routinely eliminates at will and in inappropriate places without handler command. Attempts to establish a predictable elimination schedule have failed and the dog shows no improvement with age.

39. **SOCIALLY INAPPROPRIATE BEHAVIOR WITH PEOPLE –** Exhibits poor social manners with people

Absent – Is socially pleasant around people of all ages in all settings as evidenced by not jumping on people, mouthing hands and/or muzzling.

Very Mild – Very occasionally exhibits very mild socially inappropriate behavior around people such as brief attempts to jump on people, lightly mouth hands and/or soft muzzling. Is not persistent and readily responds to redirection from handler.

Mild – Occasionally exhibits mild socially inappropriate behavior around people such as brief jumping on people, moderately mouthing hands and/or moderate muzzling. May be mildly persistent but readily responds to redirection from handler.

Mod – Frequently exhibits mildly socially inappropriate behavior or exhibits moderate socially inappropriate behavior around people such as forceful jumping on people, hard mouthing of hands and/or mild muzzle punches. May be moderately persistent requiring repeated redirection from the handler.

Severe – Exhibits severe socially inappropriate behavior around people such as forceful jumping on people, hard mouthing of hands with or without teeth and/or forceful muzzle punches. May be very persistent Responds poorly to direction from handler.

40. **INCONSISTENT –** Dog's confidence, demeanor and/or ability to cope is inconsistent and not as expected based on the recent observations and training or socialization provided.

Absent – Dog's confidence, demeanor and/or ability to cope is very consistent over multiple assessments in various environments.

Very mild – Dog's confidence, demeanor and/or ability to cope varies very mildly over multiple assessments in various environments.

Mild – Dog's confidence, demeanor and/or ability to cope varies mildly over multiple assessments in various environments.

Moderate – Dog's confidence, demeanor and/or ability to cope varies moderately over multiple assessments in various environments.

Severe – Dog's confidence, demeanor and/or ability to cope is extremely unpredictable across multiple assessments in various environments.

41. **HANDLER/DOG TEAM -** How well dog and handler work together

Work Well – Team is working well together in most situations.

– Team works well together except for brief periods in new or very stimulating situations.

– Team works well together except occasionally for moderately long periods in new or very stimulating situations.

– Effective teamwork is interrupted for moderately prolonged periods in new and/or mild to moderately stimulating situations.

Not Working Well – Effective teamwork is interrupted for extended periods in new and/or mild to moderately stimulating situations.

42. **RELATIONSHIP SKILLS** - Handler's ability to help team work well together and/or advance the dog's confidence and/or skills

9-8-7 Handler is very skilled in building a trusting relationship and advancing the dog's confidence and/or skills. Handler communicates clearly, uses appropriate praise and reward and provides clear boundaries, uses skills to direct the dog.

6-5-4 Handler is less skilled or lacks skills in some key areas, which occasionally impairs their ability to work well together and/or advance the dog's confidence and/or skills. The level of **handler** skills mildly impacts the handler's ability to build a trusting relationship with the dog, communicate clearly, use appropriate praise and reward, provide clear boundaries and use skills to direct the dog.

3-2-1 Handler is not very skilled - Handler lacks skills in some or many key areas, which frequently impairs their ability to work well together and/or advance the dog's confidence and/or skills. The lack of handler skills moderately to severely impacts the handler's ability to build a trusting relationship with the dog, communicate clearly, use appropriate praise and reward, provide clear boundaries and use skills to direct the dog.

43. **COMPARISON 9 to 1 SCORE** – This comparison score really helps us to get an overall picture of each dog. Use the 1 - 9 numerical score to grade each dog as an approximation of how well suited THIS dogs appears to be, at THIS time, related to the dogs in YOUR program, at THIS time. No need to consider how another program may rate the dog,how well suited for another line or work, or how the dog compares to the ultimate ideal dog. Use the whole range of the 1 - 9 scale where dogs fit the definition.

9-8-7 – Flexible dog, adaptable placement
This dog is really nice for our work

6-5-4 – Less adaptable placement

3-2-1 – Unlikely to be usable; limited placement options
This dog is poorly suited for our work

44. SOCIALLY INAPPROPRIATE BEHAVIOR WITH DOGS

Absent – Is socially appropriate around dogs in all settings as evidenced by not body slamming, humping, roughly pursuing play despite other dog's communications to stop.

Very Mild – Very occasionally exhibits very mild socially inappropriate behavior around dogs as evidenced by very mildly pursuing play despite other dog's communications to stop.

Mild – Occasionally exhibits mild socially inappropriate behavior around dogs as evidenced by mildly pursuing play and/or brief muzzling or mouthing despite other dog's communications to stop.

Moderate – Frequently exhibits moderate socially inappropriate behavior around dogs as evidenced by pursuing play despite other dog's communications to stop, muzzle punches, mouthing and/or brief humping.

Severe – Frequently exhibits severe socially inappropriate behavior around dogs as evidenced by persistent play despite other dog's communications to stop and/or hard muzzle punches, rough mouthing and/or persistent humping.

45. THUNDER REACTION

Absent – No change in dog's demeanor just prior to, during or immediately after a thunderstorm.

Very Mild – Very mild awareness of thunderstorm by stopping activity and/or turning attention to effects of thunderstorm for a few seconds then resumes normal behavior; does not interfere with work.

Mild – Awareness and mild anxiety of thunderstorm; exhibits mild panting and/or restlessness before and/or during the storm; does not interfere with work or response to play or known commands.

Moderate – Awareness and moderate anxiety of thunderstorm; exhibits moderate panting and/or restlessness before and/or during the storm; interferes mildly with work or response to play or known commands.

Severe – Awareness and severe anxiety of thunderstorm; exhibits severe panting and/or restlessness before, during and/or after the storm; interferes moderately or severely with work or response to play or known commands.

46. KENNELS POORLY – Adapts poorly to living in a kennel environment, evidenced by stressed emotional state and/or difficulty maintaining physical condition due to kenneling

Absent – Adapts well to living in the kennel. May be excited to see people or dogs, but does not exhibit changes in behavior typically related to poor adaptability of being kenneled such as anxious or unsettled behavior.

Very mild – Exhibits very mild changes in behavior such as occasional vocalizing, mild increased or decreased activity when kenneled and adapts within a few days to kenneling.

Mild – Exhibits mild changes in behavior such as frequent vocalizing, increased or decreased activity when kenneled. Adapts to kenneling within 2 weeks.

Moderate – Even after kenneling for 2 weeks, exhibits moderate changes in behavior such as frequent signs of inability to adapt to kenneling requiring exceptional animal husbandry or enrichment measures to remain in the kennel while maintaining health and/or calm emotional state.

Severe – Even after kenneling for 2 weeks, exhibits persistent signs of inability to adapt to kenneling and must be removed from the kennel environment to maintain health and/or calm emotional state

47. Working Speed- pace at which the dog is walking while guiding or other work the dog is trained to do.

Behavior Checklist	GPS Speed	Description
Slow	<2.5 (<4 kph)	Very slow walk
Mod-	2.5 to 2.7 (4.0-4.4 kph)	Leisurely walk
Mod	2.8 to 3.3 (4.5-5.4 kph)	Average walking speed
Mod+	3.4 to 3.7 (5.5-6.0 kph)	Walking with purpose
Fast	>3.7 (>6.0 kph)	Fast walking, just before a slow jog

48. Gait when moving out – How easily the dog maintains a trot when moving quickly.

Trots easily and maintains trot (score 1)
Paces and trots, can't maintain a trot (score 2)
Paces mostly, difficulty trotting (score 3)

49. Housebreaking problems- Frequency at which dog eliminates in the home or when confined to a crate assuming the dog is given appropriate opportunity to eliminate and does not have a medical condition impacting the dog's ability to control elimination under normal circumstances.

Absent – Never eliminates in the home or crate

Very mild – Very rarely eliminates in the home or crate and the tendency to do so has improved substantially and is unlikely to present a problem in the future. Rarely is no more often than once in 3 month for dogs 4 months and older

Mild – Occasionally eliminates in the home or crate and the tendency to do so has improved substantially and is unlikely to present a problem in the future. Greater than once every three months but no more than once a month for dogs 4 months and older

Moderate – Often eliminates in the home or crate. The dog is unreliable unless closely managed. 2-4 times a month for dogs 4 months and older

Severe – Routinely eliminates in the home or crate. The dog is unreliable and attempts to establish a predictable elimination schedule have failed. More often than 4 times a month for dogs 4 months and older.

50. Innate desire to work- Lacks intrinsic drive and motivation to learn tasks and be engaged with the handler.

Absent – Strong intrinsic eagerness and motivation to learn tasks and be engaged with the handler.

Very mild – Very mild lack of intrinsic eagerness and motivation to learn tasks and be engaged with the handler. Handler

Mild – Mild lack of intrinsic eagerness and motivation to learn tasks and be engaged with the handler.

Moderate - Moderate lack of intrinsic eagerness and motivation to learn tasks and be engaged with the handler.

Severe – Severe lack of intrinsic eagerness and motivation to learn tasks and be engaged with the handler.

51. Avoidance of exhaust from vehicles

Absent – No fear or apprehension when approaching exhaust from vehicles

Very mild – Very mild signs of avoidance when approaching exhaust from vehicles. Signs may include slight hesitancy, very mild hurrying or very mild moving away and/or mildly tense body language. Maintains work.

Mild – Mild signs of avoidance when approaching exhaust from vehicles. Signs may include mild hesitancy, mild transient compression, mild hurrying, mild moving away and/or moderately tense body language. Maintains work and improves with subsequent exposure without encouragement from handler.

Moderate – Moderate signs of avoidance when approaching exhaust from vehicles. Signs may include balking on approach, hurrying, slowing down dramatically, moderate moving away. Requires encouragement to maintain work.

Severe – Fearful when approaching exhaust from vehicles. Signs may include strong hesitancy, hurrying, balking or refusal, strong pulling way and/or very tense body language. Does not maintain work.

52. Whines excessively-

Absent- Never or very rarely briefly whines.

Very mild- Infrequently whines for brief periods, readily stops without additional support or handler intervention.

Mild- Often whines for brief periods, stops with minimal support or handler intervention.

Moderate- Whines very frequently and mildly persistently, moderate support or handler intervention will reduce whining.

Severe- Whines very frequently and very persistently, moderate support or handler intervention does not significantly reduce whining.
